# Modeling resistance to the broadly neutralizing antibody PGT121 in people living with HIV-1

**DOI:** 10.1371/journal.pcbi.1011518

**Published:** 2024-03-29

**Authors:** Tyler Cassidy, Kathryn E. Stephenson, Dan H. Barouch, Alan S. Perelson

**Affiliations:** 1 School of Mathematics, University of Leeds, Leeds, United Kingdom; 2 Center for Virology and Vaccine Research, Beth Israel Deaconess Medical Center, Boston, Massachusetts, United States of America; 3 Division of Infectious Diseases, Beth Israel Deaconess Medical Center, Boston, Massachusetts, United States of America; 4 Ragon Institute of MGH, MIT and Harvard, Cambridge, Massachusetts, United States of America; 5 Theoretical Biology and Biophysics, Los Alamos National Laboratory, Los Alamos, New Mexico, United States of America; University of California San Diego Division of Biological Sciences, UNITED STATES

## Abstract

PGT121 is a broadly neutralizing antibody in clinical development for the treatment and prevention of HIV-1 infection via passive administration. PGT121 targets the HIV-1 V3-glycan and demonstrated potent antiviral activity in a phase I clinical trial. Resistance to PGT121 monotherapy rapidly occurred in the majority of participants in this trial with the sampled rebound viruses being entirely resistant to PGT121 mediated neutralization. However, two individuals experienced long-term ART-free viral suppression following antibody infusion and retained sensitivity to PGT121 upon viral rebound. Here, we develop mathematical models of the HIV-1 dynamics during this phase I clinical trial. We utilize these models to understand the dynamics leading to PGT121 resistance and to identify the mechanisms driving the observed long-term viral control. Our modeling highlights the importance of the relative fitness difference between PGT121 sensitive and resistant subpopulations prior to treatment. Specifically, by fitting our models to data, we identify the treatment-induced competitive advantage of previously existing or newly generated resistant population as a primary driver of resistance. Finally, our modeling emphasizes the high neutralization ability of PGT121 in both participants who exhibited long-term viral control.

## Author summary

Human immunodeficiency virus (HIV)-1-specific broadly neutralizing antibodies (bnAbs) have been proposed as a novel treatment modality for the treatment and prevention of HIV-1 infection. However, bnAb monotherapy has not led to sustained viral control during treatment of HIV-1 positive individuals with viral rebound being driven by the emergence of bnAb resistance. We use mathematical models to study resistance to the V3-glycan-specific antibody PGT121 in a phase I clinical trial. We found that the level of pre-existing resistance as well as the evolutionary dynamics of PGT121 resistant and sensitive viral subpopulations drive the rebound of treatment resistant virus following a single administration of PGT121. Further, our model identifies the high neutralization potency of PGT121 as a main driver of the observed long-term ART-free viral suppression observed in two trial participants.

## Introduction

Broadly neutralizing antibodies (bnAbs) have become increasingly important in the search for a functional cure of HIV [1, 2]. A number of bnAbs have recently been tested in HIV-1 positive individuals, including anti-CD4-binding-site antibodies (VRC01 and 3BNC117) and a V3-glycan-specific antibody (10–1074) [3–6]. While these antibodies induce a transient decrease in viral load in people living with HIV (PLWH) and delay viral rebound in rheusus macaques undergoing analytic treatment interruption [4, 7], treatment with existing bnAbs has *not* led to sustained viral control. In particular, the observed viral rebound appears to occur concurrently with the emergence of antibody resistance rather than being simply due to antibody washout [4, 5, 8]. Here, we use mathematical modeling to analyse the development of resistance in a clinical trial of the monoclonal antibody PGT121 [9].

The monoclonal antibody PGT121 was isolated from an elite controller [10] and has demonstrated effectiveness in reducing SHIV levels in rhesus macques [11, 12]. PGT121 blocks viral entry by interfering with HIV binding to CD4 T-cells and was shown to effectively neutralize a majority (64%) of HIV-1 strains *in vitro* [9, 10]. A recent phase I clinical trial [Clinical trial ID:NCT02960581] tested the safety and efficacy of PGT121 in PLWH living with HIV not receiving antiretroviral therapy [9] and reported plasma viral load decay in ten of 13 participants. In eight of the ten participants who responded to PGT121, viral rebound occurred by 28 days post treatment with the rebound virus demonstrating resistance to PGT121 in *in vitro* neutralization assays. Conversely, two individuals exhibited sustained viral control lasting over 168 days post treatment. In these two participants, the rebound viruses retained partial or full sensitivity to the antibody after viral rebound [9], further suggesting the role of resistance in treatment failure in the remaining study participants who did not exhibit long-term viral control. To further elucidate the role of resistance in PGT121 failure, we study different mechanisms by which resistance either through pre-existing or emergence of resistant subpopulations, may occur using mathematical models.

Mathematical models have been used extensively to understand the dynamics of HIV infection [13–20]. In fact, computational models were recently used to understand optimal combination therapies of bnAbs [21, 22] and such combination therapies have been tested in the clinic [23]. Here, we use mathematical modeling to understand the interplay between antibody potency and time to viral rebound, as well as to study the mechanisms underlying the evolution of resistance to PGT121. In short, we develop three mathematical models that incorporate increasing levels of biological realism to understand the clinical data from the PGT121 trial [9]. After fitting each mathematical model to the *in vivo* data, we use a combination of the Bayesian Information Criteria (BIC) and biological considerations to select the most appropriate mathematical model and to identify the biological mechanisms driving the development of resistance. In particular, we identify the role of PGT121 treatment in reducing competitive suppression of a resistant viral population in the eventual viral rebound in most participants. However, for the two participants who sustained viral control long after treatment, our results suggests that high sensitivity to PGT121 led to sustained viral suppression, and our results underline the importance of the reservoir of latently infected cells in driving viral rebound for these controller participants. Finally, by examining the relationships between model parameters and treatment outcomes, we identify physiological processes that determine treatment efficacy.

## Methods

### Ethics statement

This research was approved by the Los Alamos National Laboratory Human Subjects Research Review Board.

### Viral load data

In a multicenter, open label clinical trial [Clinical trial ID: NCT02960581], 13 PLWH not receiving antiretroviral therapy (ART) were administered a single dose of 30 mg/kg of the bnAb PGT121. At two of the three centers, viral load was measured using the real-time HIV-1 assay with a lower level of quantification (LLoQ) of 40 copies/mL. At the remaining center, viral load was quantified by either the COBAS ampliPrep/COBAS TaqMan HIV-1 test (LLoQ = 23 copies/mL) or the Aptima HIV-1 Quand Assay (LLoQ = 32 copies/mL) [9].

The 13 participants were stratified by initial viral load into a high viral load group (*n* = 9, median viral load 21, 040 copies/mL) and a low viral load group (*n* = 4, median viral load 270 copies/mL). These participant groups were further characterized by pre-existing resistance to PGT121 through *in vitro* neutralization assays. In these *in vitro* assays, pseudoviruses were constructed following single genome amplification of circulating viral strains and the neutralization effect of the bnAb on these pseudoviruses was measured via a TZM-b1 assay. Virus sampled from participants in the low viral load group showed no pre-existing resistance to PGT121 *in vitro*, while the high viral load group was further subdivided into resistant and sensitive groups based on initial sensitivity to PGT121 *in vitro*. Initially, 4/9 participants in the high VL group were classified as sensitive to PGT121, while two additional participants (PTID: 2319 and 2990) were classified as partially sensitive to PGT121 at baseline [9].

**Fig 1:**
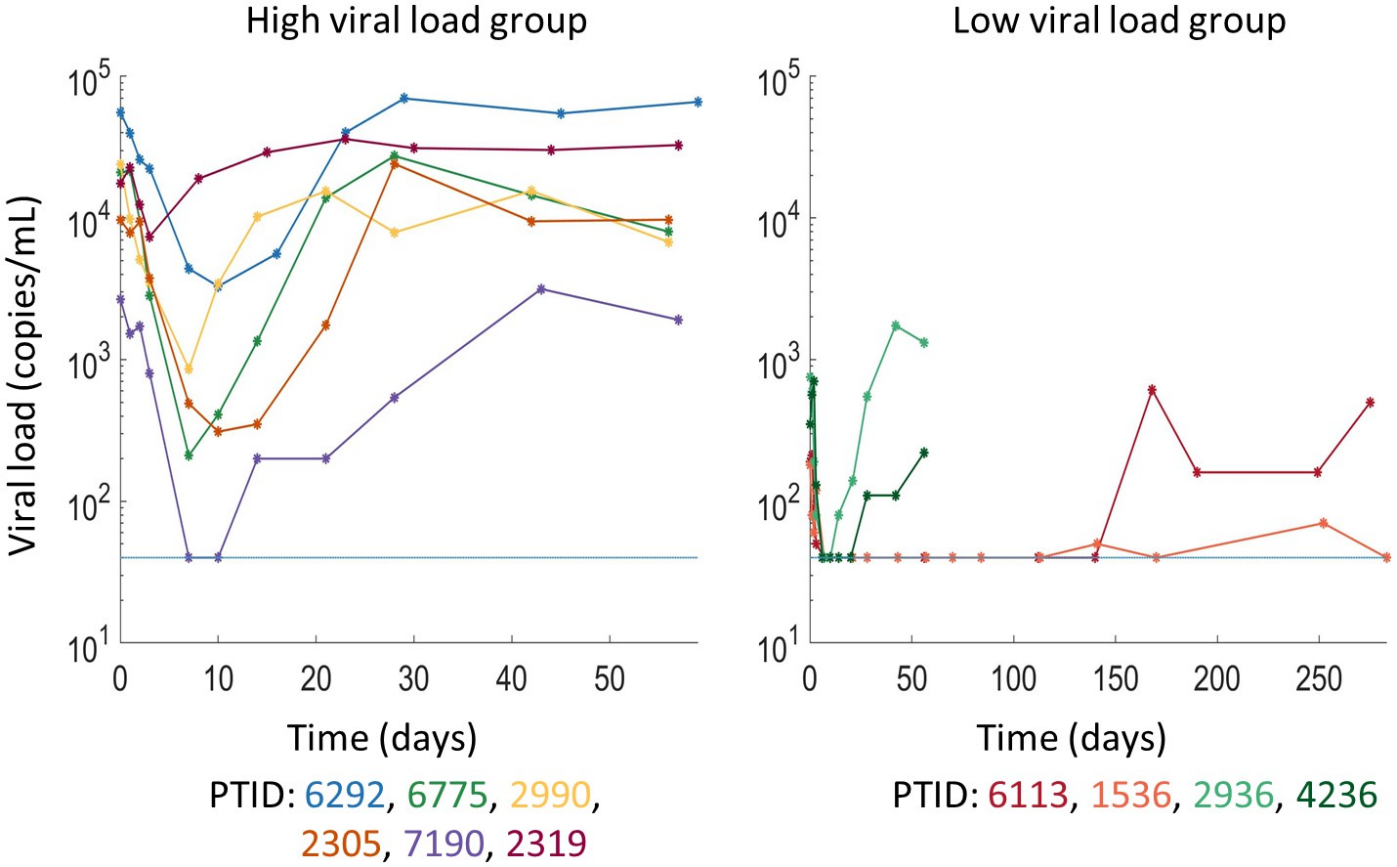
Viral load data from the 10 participants considered for modeling. Viral load concentrations shown following PGT121 infusion in participants in the Phase I trial [9] stratified into the high and low viral load groups. PGT121 was administered at day 0 and the dotted line indicates the LLoQ.

In all 8 participants with initial full sensitivity to PGT121, plasma viral load decayed following administration of PGT121. Viral dynamics were similar among these 8 participants, with a delay of approximately 1 day between PGT121 administration and the beginning viral load decay. The median viral load decrease in the high viral load group was 1.77 log among the 4 sensitive participants while all four low viral load participants had their viral load decrease to an undetectable level following therapy. In two participants with high viral load and partial sensitivity to PGT121 in pre-treatment *in vitro neutralization assays* (PTID: 2319 and 2990), the viral load also decreased during therapy. We included these two additional participants in our modeling. Viral rebound occurred within 28 days in 8/10 participants who showed viral load decline following PGT121 administration and the rebound virus was resistant to PGT121. However, two participants maintained viral load below the limit of quantification until until at least day 112, when PGT121 levels were undetectable, with sustained viral rebound occurring only after day 168 or day 252. In both these participants, the rebound viruses retained full or partial sensitivity to PGT121 [9]. We show the viral load data in Fig 1.

### Pharmacokinetics and pharmacodynamics of PGT121

The pharmacokinetics of PGT121 was measured in all 13 study participants. The concentration of PGT121 measured in serum decayed in a biphasic manner with elimination half-life estimates between 12.5 and 14 days for the 13 participants not receiving ART [9]. The lower limit of quantification of the antibody assay was 0.71 *μ*g/mL for HIV-infected participants. There were no measured anti-PGT121 antibodies prior to therapy, although two participants did develop anti-PGT121 antibodies during treatment. However, these antidrug antibodies were non-neutralizing [9].

We use a two compartment pharmacokinetic (PK) model to analyze the biphasic decay of circulating PGT121. While developing a more mechanistic model of PGT121 PK is possible, we used a simple PK model that is capable of representing the dynamics of PGT121 with model fits shown in Fig A of S1 Text. The first compartment represents the blood, with *A*_1_(*t*) being the concentration of PGT121 in plasma, while the second compartment is the peripheral tissues. We assume that PGT121 transits from the circulation into the peripheral compartment at a rate *k*_12_. Antibody in the peripheral compartment either returns to the circulation at a rate *k*_21_ or is cleared at a rate *k*_0_. We denote the volumes of each compartment by *vol*_1_ and *vol*_2_, respectively. Study participants received 30 mg/kg of body weight of PGT121 intravenously over a period of about 60 minutes. Therefore, we model the administration of PGT121 as

$$\begin{array}{c} Dose\left(t\right)=\{\begin{array}{ccc} \frac{A_{max}}{T_{inf}} & \text{if} & t<T_{inf}\\ 0 & \text{if} & t⩾T_{inf} \end{array} \end{array}$$

where *T*_*inf*_ = 1/24 days is the infusion time, and *A*_*max*_ is the maximal measured antibody concentration. The two compartment PK model is then

$$\begin{array}{c} \begin{array}{c} \frac{d}{\text{dt}}A_{1}\left(t\right)=Dose\left(t\right)-k_{12}A_{1}\left(t\right)+\frac{vol_{2}}{vol_{1}}k_{21}A_{2}\left(t\right)\\ \frac{d}{\text{dt}}A_{2}\left(t\right)=\frac{vol_{1}}{vol_{2}}k_{12}A_{1}\left(t\right)-k_{21}A_{2}\left(t\right)-k_{0}A_{2}\left(t\right), \end{array}\} \end{array}$$ (1)

where the ratio of volumes in Eq (1) represents the different compartment volumes. While we do not know the distribution of PGT121 in the peripheral compartment, we assume that antibody transport is balanced between compartments and enforce the relationship between the transit rates

$$\begin{array}{c} k_{21}vol_{2}=k_{12}vol_{1}. \end{array}$$

Then, setting *vol*_1_ = 3L of plasma, we can calculate antibody distribution between both compartments. We note that, by discounting transit to the tissues during PGT121 administration and approximating Eq (1) by

$$\begin{array}{c} \frac{d}{\text{dt}}A_{1}\left(t\right)=Dose\left(t\right)\;\text{for}\;t\in \left[0,T_{inf}\right], \end{array}$$

it is possible to calculate *A*_1_(*t*) independently of the ratio of the compartment volumes *vol*_1_/*vol*_2_ [24]. In Fig F of S1 Text, we compare the fits of the full PK model (1) to the approximation of *A*_1_(*t*) obtained by discounting PGT121 transit to the peripheral tissues during administration.

The simplest mechanism to explain viral load decay following administration of PGT121 is antibody mediated neutralization of infectious particles. We assume a Hill coefficient of 1 and denote the half effect concentration by *EC*_50_, so PGT121 decreases the infection rate *β* by an antibody dependent factor

$$\begin{array}{c} (1-\frac{A_{1}\left(t\right)}{EC_{50}+A_{1}\left(t\right)})=\frac{1}{1+\alpha A_{1}\left(t\right)}. \end{array}$$

where *α* = 1/*EC*_50_.

As PGT121 needs to distributed throughout the body and bind the envelope protein of HIV in order to neutralize it, there may be a delay *τ* between PGT121 administration and the beginning of viral decay. Therefore, we follow [24] and model the effect of PGT121 on the infection rate *β* by

$$\begin{array}{c} \hat{\beta }\left(t\right)=\{\begin{array}{ccc} \beta & \text{if} & t<\tau \\ \frac{\beta }{1+\alpha A(t)} & \text{if} & t⩾\tau . \end{array} \end{array}$$ (2)

We note that in this model for the pharmacodynamics of PGT121, the proportion of antibody cleared between *t* = 0 and *t* = *τ* has no clinical effect.

### Viral dynamic model

**Fig 2:**
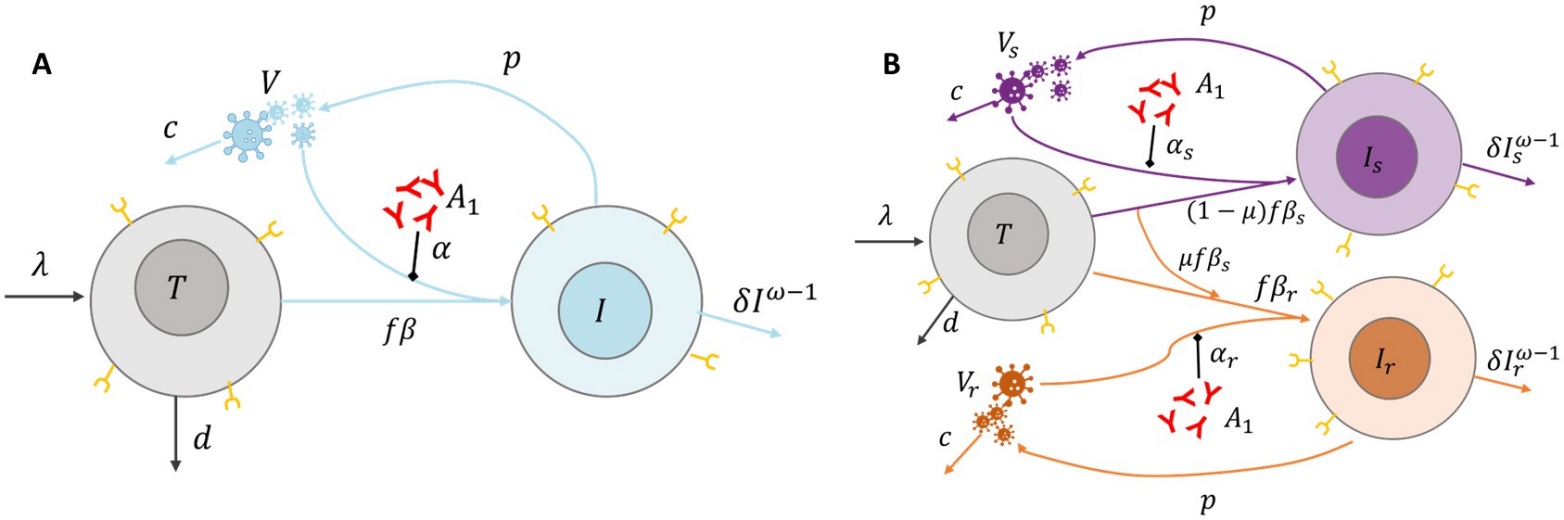
Schematics of the mathematical models of PGT121 neutralization and resistance. Panel **A** illustrates the single viral population model in Eq (5). Target cells *T* are produced at a constant rate λ and cleared linearly at rate *d*. Following contact with a virus particle *V*, target cells are productively infected at rate *fβV* and become infected cells *I*. Infected cells are cleared non-linearly with rate *δI*^*ω*−1^ and produce free virus at a per capita rate *p*. Free virus is cleared linearly at rate *c*. Panel **B** illustrates the two viral population models given in Eqs (6) and (7). Here, a proportion (1 − *μ*) of productive infections by sensitive virus creates sensitive infected cells, *I*_*s*_. The remaining proportion of these infections, *μ*, generate the resistant infected cell population *I*_*r*_ due to mutation during reverse transcription. Productive infection with a resistant virus also produces resistant infected cells. Both classes of infected cells are cleared non-linearly with rates $\delta I_{s}^{\omega -1}$ and $\delta I_{r}^{\omega -1}$, and produce virus particles at a per capita rate *p*. Free virus is cleared linearly at rate *c*. In both cases, plasma PGT121 (*A*_1_) acts to block infection.

We use three distinct mathematical models to explore the mechanisms responsible for viral load decline following administration of PGT121. In particular, we are interested in quantifying the role of pre-existing or emergent resistance to PGT121. Following [24], we adapt the basic model of viral dynamics [19, 20, 25] to account for the neutralization effect of the antibody, the development of resistance, and study antibody induced viral control. Schematic diagram of the models considered in this work are in Fig 2.

The standard viral dynamic model tracks the time evolution of target cells, *T*(*t*), infected cells, *I*(*t*), and viral particles, *V*(*t*), in time. Variants of this model have been extensively used to understand the effect of ART, the role of the immune system in viral control, and the mechanisms underlying viral rebound following reactivation of latently infected cells [15, 17]. In the standard mathematical model, target cells are produced at a constant rate λ, are cleared linearly at a rate *d*, and become infected at a rate *β* after contact with a viral particle. Productively infected cells are cleared at constant rate *d*_*I*_ and produce viral particles with rate *p*. These virions are cleared at rate *c* per day. The standard model of viral dynamics is therefore given by

$$\begin{array}{c} \begin{array}{c} \frac{d}{\text{dt}}T\left(t\right)=\lambda -\beta T(t)V(t)-dT(t)\\ \frac{d}{\text{dt}}I\left(t\right)=\beta T\left(t\right)V\left(t\right)-d_{I}I\left(t\right)\\ \frac{d}{\text{dt}}V\left(t\right)=pI(t)-cV(t). \end{array}\} \end{array}$$ (3)

However, it has been suggested that assuming linear clearance of infected cells may over simplify immune involvement, and that the clearance rate should be non-constant [26]. Holte et al. proposed one of the simplest adaptations to Eq (3) to include density dependent clearance of infected cells by setting *d*_*I*_ = *δI*^*ω*−1^. There are a number of phenomenological explanations for the non-linear term *I*^*ω*−1^, including a non-linear relationship between immune effector cells and infected cell densities [26, 27]. Further, only a fraction *f* of infection events produce productively infected cells due to abortive infection [24, 28–30]. Including these effects in Eq (3) gives the baseline model for the remainder of our study

$$\begin{array}{c} \begin{array}{c} \frac{d}{\text{dt}}T\left(t\right)=\lambda -\beta T(t)V(t)-dT(t)\\ \frac{d}{\text{dt}}I\left(t\right)=f\beta T\left(t\right)V\left(t\right)-\delta {\left[I\left(t\right)\right]}^{\omega }\\ \frac{d}{\text{dt}}V\left(t\right)=pI(t)-cV(t). \end{array}\} \end{array}$$ (4)

We note that by setting *ω* = 1, *f* = 1, and *δ* = *d*_*I*_ in Eq (4) becomes the basic viral dynamic model in Eq (3) with abortive infection. Thus, it is simple to test if the inclusion of the density dependent clearance of immune cells is necessary to increase the biological relevance of the mathematical model when compared against the viral dynamics data.

#### Delayed neutralization

The simplest viral dynamic model we consider incorporates delayed neutralization in the basic model of viral dynamics, Eq (3), and is given by

$$\begin{array}{c} \begin{array}{c} \frac{d}{\text{dt}}T\left(t\right)=\lambda -\hat{\beta }\left(t\right)T\left(t\right)V\left(t\right)-dT\left(t\right)\\ \frac{d}{\text{dt}}I\left(t\right)=f\hat{\beta }\left(t\right)T\left(t\right)V\left(t\right)-\delta {\left[I\left(t\right)\right]}^{\omega }\\ \frac{d}{\text{dt}}V\left(t\right)=pI(t)-cV(t) \end{array}\} \end{array}$$ (5)

where $\hat{\beta }\left(t\right)$ is given in Eq (2) and accounts for the antibody mediated neutralization of viral particles.

#### Resistant population model

Viral sequencing and *in vitro* studies show that the rebound viral population in most participants carry viral strains that are resistant to PGT121. Accordingly, we adapt Eq (5) to include a subpopulation of virus that is resistant to PGT121. While structuring the viral load into two compartments is a simplification of the viral diversity present in the participants, it allows for a simple study of the role of PGT121 resistance in driving viral rebound and is common in viral dynamic models of therapeutic resistance in HIV [24, 31, 32]. In this sense, we emphasize that we do not attempt to characterise the myriad of pathways to PGT121 resistance, nor the genetic heterogeneity present in HIV but rather attempt to study the role of a resistant subpopulation of virus in driving viral rebound.

The total viral concentration *V*(*t*) is the sum of the sensitive, *V*_*s*_(*t*), and resistant, *V*_*r*_(*t*), subpopulations, given by *V*(*t*) = *V*_*s*_(*t*) + *V*_*r*_(*t*). We assume that the dynamics of *V*_*s*_(*t*) and *V*_*r*_(*t*) are similar, but with population specific infectivity rates, *β*_*s*_ and *β*_*r*_, and neutralization effect of PGT121, *α*_*s*_ and *α*_*r*_, respectively. Then, during treatment, the population specific infection rates are given by

$$\begin{array}{c} {\hat{\beta }}_{s}\left(t\right)=\{\begin{array}{ccc} \beta_{s} & \text{if} & t<\tau \\ \frac{\beta_{s}}{1+\alpha_{s}A\left(t\right)} & \text{if} & t⩾\tau , \end{array}\;\text{and}\;{\hat{\beta }}_{r}\left(t\right)=\{\begin{array}{ccc} \beta_{r} & \text{if} & t<\tau \\ \frac{\beta_{r}}{1+\alpha_{r}A\left(t\right)} & \text{if} & t⩾\tau . \end{array} \end{array}$$

We also structure the infected cells to distinguish between cells infected by the sensitive and resistant viral populations. We denote the total concentration of infected cells by *I*(*t*) = *I*_*s*_(*t*) + *I*_*r*_(*t*) where *I*_*s*_(*t*) and *I*_*r*_(*t*) are the concentrations of cells infected by the PGT121 sensitive and resistant populations, respectively. We do not consider viral mutation in this model and therefore assume that infected cells produce viral particles with the same sensitivity to PGT121 as the virus which infected the cell. The resulting two population viral dynamic model is

$$\begin{array}{c} \begin{array}{c} \frac{d}{\text{dt}}T\left(t\right)=\lambda -{\hat{\beta }}_{s}\left(t\right)T\left(t\right)V_{s}\left(t\right)-{\hat{\beta }}_{r}\left(t\right)T\left(t\right)V_{r}\left(t\right)-dT\left(t\right)\\ \frac{d}{\text{dt}}I_{s}\left(t\right)=f\hat{\beta_{s}}\left(t\right)T\left(t\right)V_{s}\left(t\right)-\delta {\left[I_{s}\left(t\right)\right]}^{\omega }\\ \frac{d}{\text{dt}}V_{s}\left(t\right)=pI_{s}\left(t\right)-cV_{s}\left(t\right)\\ \frac{d}{\text{dt}}I_{r}\left(t\right)=f\hat{\beta_{r}}\left(t\right)T\left(t\right)V_{r}\left(t\right)-\delta {\left[I_{r}\left(t\right)\right]}^{\omega }\\ \frac{d}{\text{dt}}V_{r}\left(t\right)=pI_{r}\left(t\right)-cV_{r}\left(t\right). \end{array}\} \end{array}$$ (6)

#### Mutation driven resistance

The two population model in Eq (6) assumes that the resistant population is present prior to treatment, and that there is no adaptation to the selection pressure against the sensitive population. To test if mutation from sensitive to resistant viral populations is a driver of the observed resistance, we include mutation from the sensitive population to the resistant population in Eq (7). We denote the mutation rate by *μ*, and assume that viral mutation occurs during infection due to mutations during reverse transcription. To simplify the model, we discount mutation from the resistant population back to the sensitive population. The resulting mathematical model is

$$\begin{array}{c} \begin{array}{c} \frac{d}{\text{dt}}T\left(t\right)=\lambda -{\hat{\beta }}_{s}\left(t\right)T\left(t\right)V_{s}\left(t\right)-{\hat{\beta }}_{r}\left(t\right)T\left(t\right)V_{r}\left(t\right)-dT\left(t\right)\\ \frac{d}{\text{dt}}I_{s}\left(t\right)=f\left(1-\mu \right)\hat{\beta_{s}}\left(t\right)T\left(t\right)V_{s}\left(t\right)-\delta {\left[I_{s}\left(t\right)\right]}^{\omega }\\ \frac{d}{\text{dt}}V_{s}\left(t\right)=pI_{s}\left(t\right)-cV_{s}\left(t\right)\\ \frac{d}{\text{dt}}I_{r}\left(t\right)=f\hat{\beta_{r}}\left(t\right)T\left(t\right)V_{r}\left(t\right)+f\mu \hat{\beta_{s}}\left(t\right)T\left(t\right)V_{s}\left(t\right)-\delta {\left[I_{r}\left(t\right)\right]}^{\omega }\\ \frac{d}{\text{dt}}V_{r}\left(t\right)=pI_{r}\left(t\right)-cV_{r}\left(t\right). \end{array}\} \end{array}$$ (7)

While the mutation rate *μ* is not precisely known, it has been estimated as approximately 3 × 10^−5^ [33–35]. Here, we do not consider insertion and deletion mutations and therefore set *μ* = 2.16 × 10^−5^ [36], although some recent work indicates that it may be much higher [37].

#### Latent reactivation and viral control

Two participants exhibited long-term viral control following PGT121 washout, although viral rebound ultimately occurred. In these participants, it is possible that reactivation of latently infected cells led to viral rebound. Thus, we included a population of latently infected cells, *L*(*t*). While it is possible to use an explicit model for the concentration of these latently infected cells [13, 15, 20, 38, 39], the half life of the latent reservoir has been estimated as at least 44 months [40–44], significantly longer than the time to viral rebound observed in the PGT121 trial. Thus, we assume that the concentration of latently infected cells is approximately constant during the study and set *L*(*t*) = *L*_0_. Assuming that latently infected cells reactivate at a constant rate *a*, we obtain the reactivation term *aL*_0_ and derive bounds for *a* in S1 Text [14, 45]. We emphasize that the virus was sensitive to PGT121 at baseline in both long-term controllers and therefore we assume that any re-activated latent cells were likely generated prior to treatment and will thus also be PGT121 sensitive. We include latent cell reactivation in the viral dynamic model when considering the two long-term controllers through a constant production rate of sensitive infected cells *I*_*s*_(*t*). Thus, the single strain mathematical model in Eq (5) becomes

$$\begin{array}{c} \begin{array}{c} \frac{d}{\text{dt}}T\left(t\right)=\lambda -\hat{\beta }T\left(t\right)V\left(t\right)-dT\left(t\right)\\ \frac{d}{\text{dt}}I\left(t\right)=aL_{0}+f\hat{\beta }\left(t\right)T\left(t\right)V\left(t\right)-\delta {\left[I\left(t\right)\right]}^{\omega }\\ \frac{d}{\text{dt}}V\left(t\right)=pI(t)-cV(t) \end{array}\} \end{array}$$ (8)

while the two strain models in Eqs (6) and (7) become

$$\begin{array}{c} \begin{array}{c} \frac{d}{\text{dt}}T\left(t\right)=\lambda -{\hat{\beta }}_{s}\left(t\right)T\left(t\right)V_{s}\left(t\right)-{\hat{\beta }}_{r}\left(t\right)T\left(t\right)V_{r}\left(t\right)-dT\left(t\right)\\ \frac{d}{\text{dt}}I_{s}\left(t\right)=aL_{0}+f\hat{\beta_{s}}\left(t\right)T\left(t\right)V_{s}\left(t\right)-\delta {\left[I_{s}\left(t\right)\right]}^{\omega }\\ \frac{d}{\text{dt}}V_{s}\left(t\right)=pI_{s}\left(t\right)-cV_{s}\left(t\right)\\ \frac{d}{\text{dt}}I_{r}\left(t\right)=f\hat{\beta_{r}}\left(t\right)T\left(t\right)V_{r}\left(t\right)-\delta {\left[I_{r}\left(t\right)\right]}^{\omega }\\ \frac{d}{\text{dt}}V_{r}\left(t\right)=pI_{r}\left(t\right)-cV_{r}\left(t\right) \end{array}\} \end{array}$$ (9)

and

$$\begin{array}{c} \begin{array}{c} \frac{d}{\text{dt}}T\left(t\right)=\lambda -{\hat{\beta }}_{s}\left(t\right)T\left(t\right)V_{s}\left(t\right)-{\hat{\beta }}_{r}\left(t\right)T\left(t\right)V_{r}\left(t\right)-dT\left(t\right)\\ \frac{d}{\text{dt}}I_{s}\left(t\right)=aL_{0}+f\left(1-\mu \right)\hat{\beta_{s}}\left(t\right)T\left(t\right)V_{s}\left(t\right)-\delta {\left[I_{s}\left(t\right)\right]}^{\omega }\\ \frac{d}{\text{dt}}V_{s}\left(t\right)=pI_{s}\left(t\right)-cV_{s}\left(t\right)\\ \frac{d}{\text{dt}}I_{r}\left(t\right)=f\hat{\beta_{r}}\left(t\right)T\left(t\right)V_{r}\left(t\right)+f\mu \hat{\beta_{s}}\left(t\right)T\left(t\right)V_{s}\left(t\right)-\delta {\left[I_{r}\left(t\right)\right]}^{\omega }\\ \frac{d}{\text{dt}}V_{r}\left(t\right)=pI_{r}\left(t\right)-cV_{r}\left(t\right). \end{array}\} \end{array}$$ (10)

### Parameter estimation

#### Pharmacokinetic parameters

For each participant in the PGT121 trial, we separately fit the two compartment pharmacokinetic model Eq (1) to the measured circulating PGT121 concentration. We fixed *vol*_1_ = 3L and fit the kinetic parameters *k*_12_, *k*_21_, and *k*_0_. We minimized the least squares error between the log_10_ of the PGT121 concentration sampled at time *t*_*i*_ and log_10_(*A*_1_(*t*_*i*_)), where *A*_1_(*t*) is the numerical solution of Eq (1). Fits of the PK model (Eq (1)) to the participant data are shown in Fig A of S1 Text and the best-fit parameters are given in Table B of S1 Text. We then use the participant specific PK parameters in the fitting and simulation of the viral dynamic models.

#### Fixed viral dynamic parameters

We held a number of parameters constant in our viral dynamic models fixed at values derived from the literature throughout the remainder of our study. These fixed parameters represent physiological processes that are presumably antibody independent, such as mechanisms intrinsic to the viral life cycle. We fixed the infection independent death rate of target cells to be *d* = 0.01 day^−1^ [46], the clearance rate of viral particles at *c* = 23 day^−1^ [47] and set the percentage of abortive infection to be 95%, so that *f* = 0.05 [28]. We follow [24] and take *δ* such that *δI*(0) = 1.5 day^−1^. The participants in the PGT121 trial were chronically infected at study enrollment with a mean CD4 T-cell concentration of 649, 500 cells/mL [9]. As CD4 T-cell counts were not reported for each individual, we fixed *T*(0) = 649, 500 cells/mL for all participants.

#### Initial conditions of the single viral population model

To determine initial conditions for the remaining model species, we assume that the viral load observed before enrolling in the trial represents the participant specific set-point viral load, corresponding to an infected equilibrium. With the initial viral load, *V*(0), taken from the participant data, we calculate

$$\begin{array}{c} I\left(0\right)=\frac{cV(0)}{p}, \end{array}$$

and choose *δ* such that 1.5 = *δ*[*I*(0)]^*ω*−1^ [24]. Then, to impose that the baseline viral load represents the set-point viral load, we calculate

$$\begin{array}{c} \beta =\frac{\delta {\left[I\left(0\right)\right]}^{\omega }}{fT(0)V(0)}=\frac{1.5I(0)}{fT(0)V(0)},\;\text{and}\;\lambda =T\left(0\right)(\beta V(0)+d). \end{array}$$

#### Initial conditions of the two population models

For the two viral population models (Eqs (6) and (7)), the proportion of the baseline viral load, *V*_0_, resistant to PGT121 is given by *ρ*. Thus, the concentration of resistant virus at baseline is *V*_*r*_(0) = *ρV*(0) while the concentration of sensitive virus given by *V*_*s*_(0) = (1 − *ρ*)*V*(0). After stratifying the baseline viral load, we calculate the initial proportion of infected cells that produce each viral population by

$$\begin{array}{c} I_{s}\left(0\right)=\frac{cV_{s}\left(0\right)}{p},\;\text{and}\;I_{r}\left(0\right)=\frac{cV_{r}\left(0\right)}{p}. \end{array}$$ (11)

Using *I*(0) = *I*_*s*_(0) + *I*_*r*_(0), we calculate the clearance rate of infected cells from *δI*(0)^*ω*−1^ = 1.5, and thus obtain

$$\begin{array}{c} \beta_{s}=\frac{\delta {\left[I_{s}\left(0\right)\right]}^{\omega }}{fT\left(0\right)V_{s}\left(0\right)},\;\beta_{r}=\frac{\delta {\left[I_{r}\left(0\right)\right]}^{\omega }}{fT\left(0\right)V_{r}\left(0\right)},\;\lambda =T\left(0\right)(\beta_{s}V_{s}\left(0\right)+\beta_{r}V_{r}\left(0\right)+d). \end{array}$$ (12)

#### Viral dynamic parameter estimation

For the mathematical models given in Eqs (5), (6) and (7), we denote the unknown parameters by *θ* and estimated these parameters by fitting the model to data from each individual participant from the PGT121 phase I trial. As we were interested in the viral load dynamics between PGT121 administration and its eventual rebound to baseline, we restricted our fitting to the first 56 days in all participants except the long-term controllers. For all participants except participant 2319, we considered the viral load on day 0 as the baseline value. For participant 2319, we obtained better model fits to the data by using the screening viral load rather than the viral load on day 0 as the baseline value.

In general, we fit the parameters *θ* for each mathematical model by minimizing the error functional corresponding to the least squares estimate,

$$\begin{array}{c} {\text{LSE}}_{j}\left(\theta \right)=\sum_{i=1}^{N}{\left[\text{log}\left(v_{j,i}\right)-\text{log}\left(V\left(t_{i},\theta \right)\right)\right]}^{2}, \end{array}$$

where *v*_*j*,*i*_ is the viral load measured from the *j*-th participant at time *t*_*i*_ and *V*(*t*_*i*_, *θ*) is the viral load sampled at time *t*_*i*_ from the numerical simulation of the corresponding mathematical model parametrized by *θ*. In models with both resistant and sensitive viral populations, we recall that *V*(*t*_*i*_) = *V*_*s*_(*t*_*i*_) + *V*_*r*_(*t*_*i*_).

For participants in the low baseline viral load group (participants 6113, 2936, 4236, and 1536), the viral load falls below the lower limit of quantification of the viral load assay, LLoQ = 40 copies/mL. To account for this data censoring, we follow [39] and [48] and assume that viral loads below the limit of quantification are log-normally distributed with mean *V*(*t*_*i*_) and variance *σ*. Then, the corresponding loglikelihood for the *j*-th participant is

$$\begin{array}{c} F_{j}\left(\theta \right)=\sum_{i\in \chi_{o}}{\left[\text{log}\left(v_{j,i}\right)-\text{log}\left(V\left(t_{i}\right)\right)\right]}^{2}-\sum_{i\in \chi_{c}}H[\text{LLoQ},\text{log}(V\left(t_{i}\right),\sigma )], \end{array}$$ (13)

where *χ*_*o*_ and *χ*_*c*_ represent the set of measurements where the viral loads that are observed or censored, respectively and

$$\begin{array}{c} H[\text{log}\left(\text{LLoQ}\right),\text{log}(V\left(t_{i}\right),\sigma )]=\text{log}(\frac{1}{\sqrt{2\pi \sigma^{2}}}\int_{-\infty }^{\;\text{log}(\text{LLoQ})}\text{exp}(\frac{1}{2\sigma^{2}}[u-\text{log}(V\left(t_{i}\right))])du) \end{array}$$

is the cumulative density function for the normal distribution. It follows that

$$\begin{array}{c} H[\text{log}\left(\text{LLoQ}\right),\text{log}(V\left(t_{i}\right),\sigma )]=\text{log}(\frac{1}{2}[1+\text{erf}(\frac{\;\text{log}\left(\text{LLoQ}\right)-\text{log}(V\left(t_{i}\right))}{\sqrt{2}\sigma })]). \end{array}$$

For participants with censored data, we also estimate the random effect of the censored data, *σ*. We note that, for participants with viral load that does not fall below the limit of detection, *χ*_*c*_ is empty so Eq (13) reduces to the least squares estimate LSE. The fit parameter θ* for the *j*-th participant is therefore determined by

$$\begin{array}{c} \theta_{j}^{*}={\text{argmin}}_{\theta }F_{j}\left(\theta \right). \end{array}$$

For the single population viral dynamics model given in Eq (5), we fit the parameters *α*, *τ*, *ω*, and *p* for each participant. For the two viral population models (Eqs (6) and (7)), we re-fit the four parameters *α*_*s*_, *τ*, *ω*, and *p*, and we assume that the resistant and sensitive viral populations differ only in their infectivity rate and response to antibody. Thus, the addition of the resistant population only requires the fitting of two additional parameters, the first representing the neutralization effect of PGT121 on the resistant subpopulation, *α*_*r*_, and the second modeling the proportion of the initial viral load that forms the PGT121 resistant subpopulation, *ρ* ∈ (0, 1).

#### Parameter identifiability analysis

We use profile likelihood based techniques to evaluate the robustness of our parameter estimates for each parameter, and denote the *i*-th component of fit parameter to the *j*-th participant $\theta_{j}^{*}$ by $\theta_{j,i}^{*}$. We used a profile likelihood approach to study the structural and practical identifiability of $\theta_{j,i}^{*}$ [49, 50]. The profile likelihood of the fit model parameter $\theta_{j,i}^{*}$ is defined by

$${\text{PL}}_{\theta_{j,i}}\left(c\right)=\underset{\theta_{j}|\theta_{j,i}=c}{\text{min}}F_{j}\left(\theta \right),$$

which is computed by fixing *θ*_*j*,*i*_ = *c*, then re-minimizing the error function *F*_*j*_(*θ*) over the remaining free parameters. Naively, the profile likelihood of *θ*_*j*,*i*_ characterizes the error functional, *F*_*j*_, profile as *θ*_*j*,*i*_ is fixed away from the optimal value for each individual [50, 51]. The re-optimization step is crucial in this step to fully characterise any non-linear relationships between parameters [51, 52]. For a given confidence degree *η*, the corresponding confidence interval is the set of values *c* that satisfy

$$\begin{array}{c} {\text{PL}}_{\theta_{j,i}}\left(c\right)-F_{LL}\left(\theta_{j}^{*}\right)<\chi_{\eta ,1}^{2} \end{array}$$

where $\chi_{\eta ,1}^{2}$ is the *η* quantile of the *χ*^2^ distribution with one degree of freedom. These confidence intervals are determined by the likelihood-ratio test for nested models, where fixing *θ*_*i*,*j*_ = *c* acts to impose a single constraint on the parameter estimation step. The profile based confidence interval can also be used to determine the structural or practical identifiability of the parameter *θ*_*j*,*i*_. Simply, the parameter *θ*_*j*,*i*_ is practically identifiable if the confidence interval is finite [49, 50, 53].

#### Local sensitivity analysis

We performed a local sensitivity analysis to understand the role of differences in parameter values in determining treatment response. We considered both the single and the two population mathematical model given by Eqs (5), (6) and (7) during the sensitivity analysis. We varied the parameters *ω*, *p*, *α*_*s*_ and *τ* by ±10% about their fit values for each participant, and measured the percent change in minimum viral load, time to rebound, and time to re-sensitization, where *time to rebound* was defined as the first time that the simulated viral load rebounded to 75% of the baseline viral load and *time to re-sensitization* was the first time that the sensitive population comprised over 50% of the total viral population post viral rebound. We note that our definition of viral rebound differs from Stephenson et al. [9] where they defined viral rebound as the first time point where the circulating viral load reaches 0.5log_10_ below the baseline viral load. To translate these individual results into population level insights, we combined the individual results by calculating the median percent change in the minimum viral load and the time to rebound for each parameter.

Further, we searched for correlations between fitted parameters to identify functional relationships between physiological processes, [54, 55]. After fitting each model to participant specific data, we computed the matrix of correlation coefficients between the fitted parameters and identified parameters that had significant correlation with *p* < 0.05.

## Results

### Model fits to participant data

**Fig 3:**
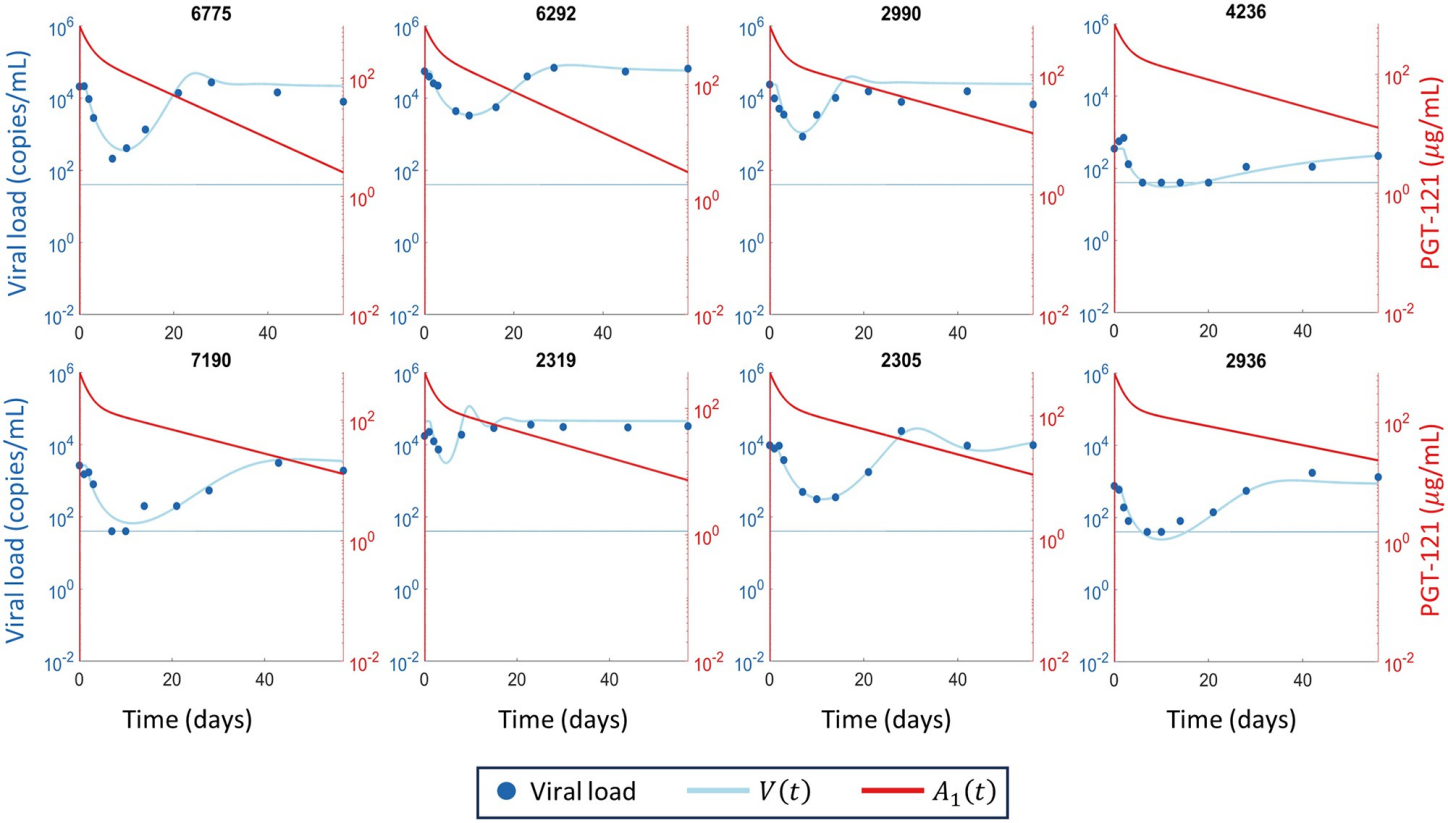
Fitting the single population mathematical model to participant data. The viral load data is shown in blue circles, the LLoQ is shown as a horizontal dotted blue line, and the simulated total viral load *V*(*t*) from the single population mathematical model Eq (5) are shown in solid blue. The simulated PGT121 concentrations are shown in solid red. The fitted parameter estimates for each participant are given in Table A of S1 Text.

**Fig 4:**
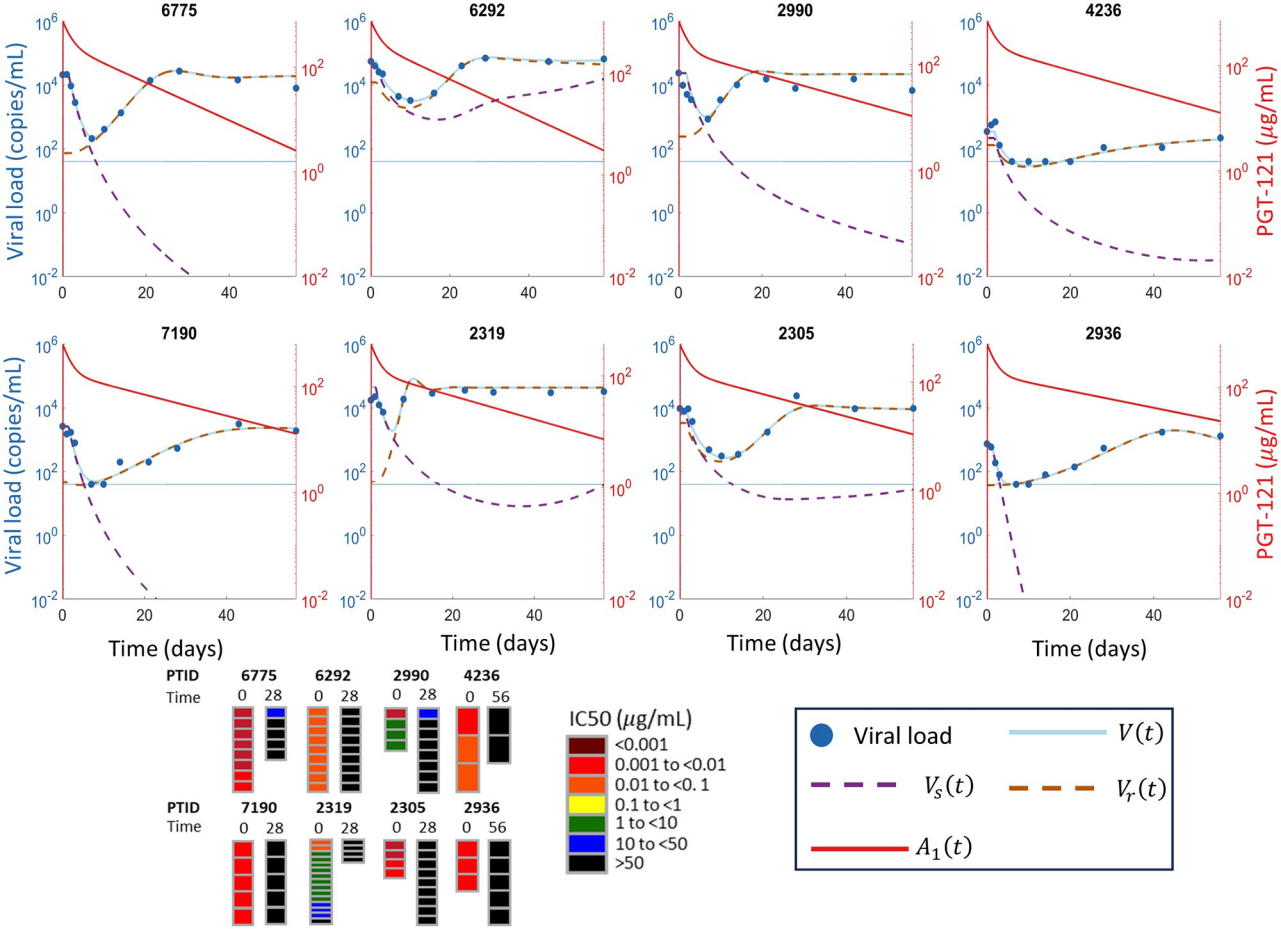
Fitting the two viral population mathematical model without mutation to participant data. The viral load data is shown in blue circles, the LLoQ is shown as a horizontal dotted blue line, and the simulated total viral load *V*(*t*) = *V*_*s*_(*t*) + *V*_*r*_(*t*) from the two-population model without mutation in Eq (6) is shown in solid blue. The PGT121 sensitive viral population *V*_*s*_(*t*) is shown in dashed purple while the resistant viral population *V*_*r*_(*t*) is shown in dashed orange. Note that the total virus and the orange dashed line denoting the resistant virus largely overlap in most panels. The simulated PGT121 concentrations are shown in solid red. The fitted parameter estimates for each participant are in Table C of S1 Text. The *in vitro* neutralization IC_50_ results from [9], with the time in days that the corresponding samples were taken, are shown at the bottom.

**Fig 5:**
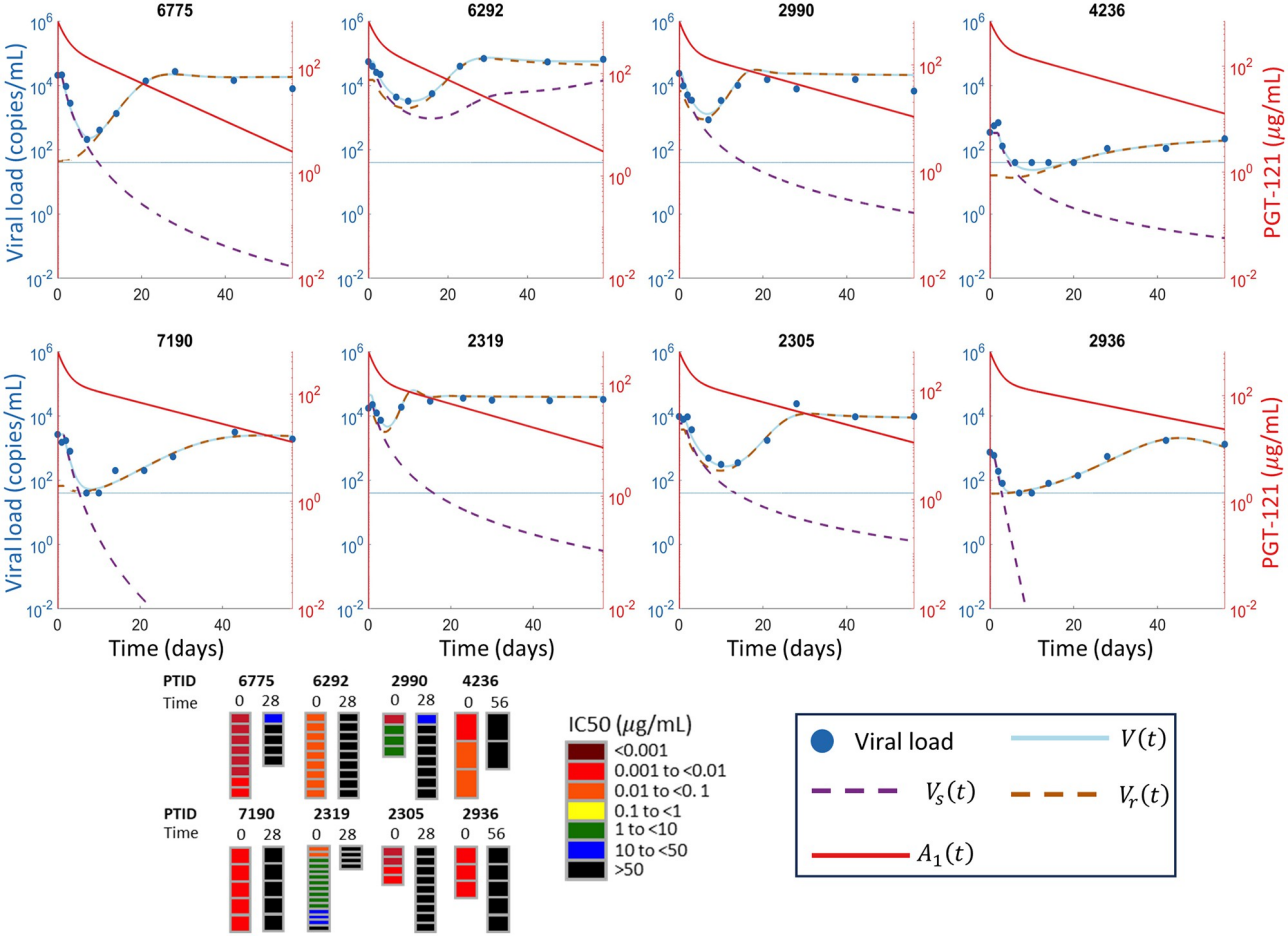
Fitting the two viral population mathematical model with mutation to participant data. The viral load data is shown in blue circles, the LLoQ is shown as a horizontal dotted blue line, and the simulated total viral load *V*(*t*) = *V*_*s*_(*t*) + *V*_*r*_(*t*) from the two-population with mutation mathematical model in Eqs (7) is shown in solid blue. The PGT121 sensitive viral population *V*_*s*_(*t*) is shown in dashed purple while the resistant viral population *V*_*r*_(*t*) is shown in dashed orange. Note that the total virus and the orange dashed line denoting the resistant virus largely overlap in most panels. The simulated PGT121 concentrations are shown in solid red. The fitted parameter estimates for each participant are in Table D of S1 Text. The *in vitro* neutralization IC_50_ results from [9], with the time in days that the corresponding samples were taken, are shown at the bottom.

**Fig 6:**
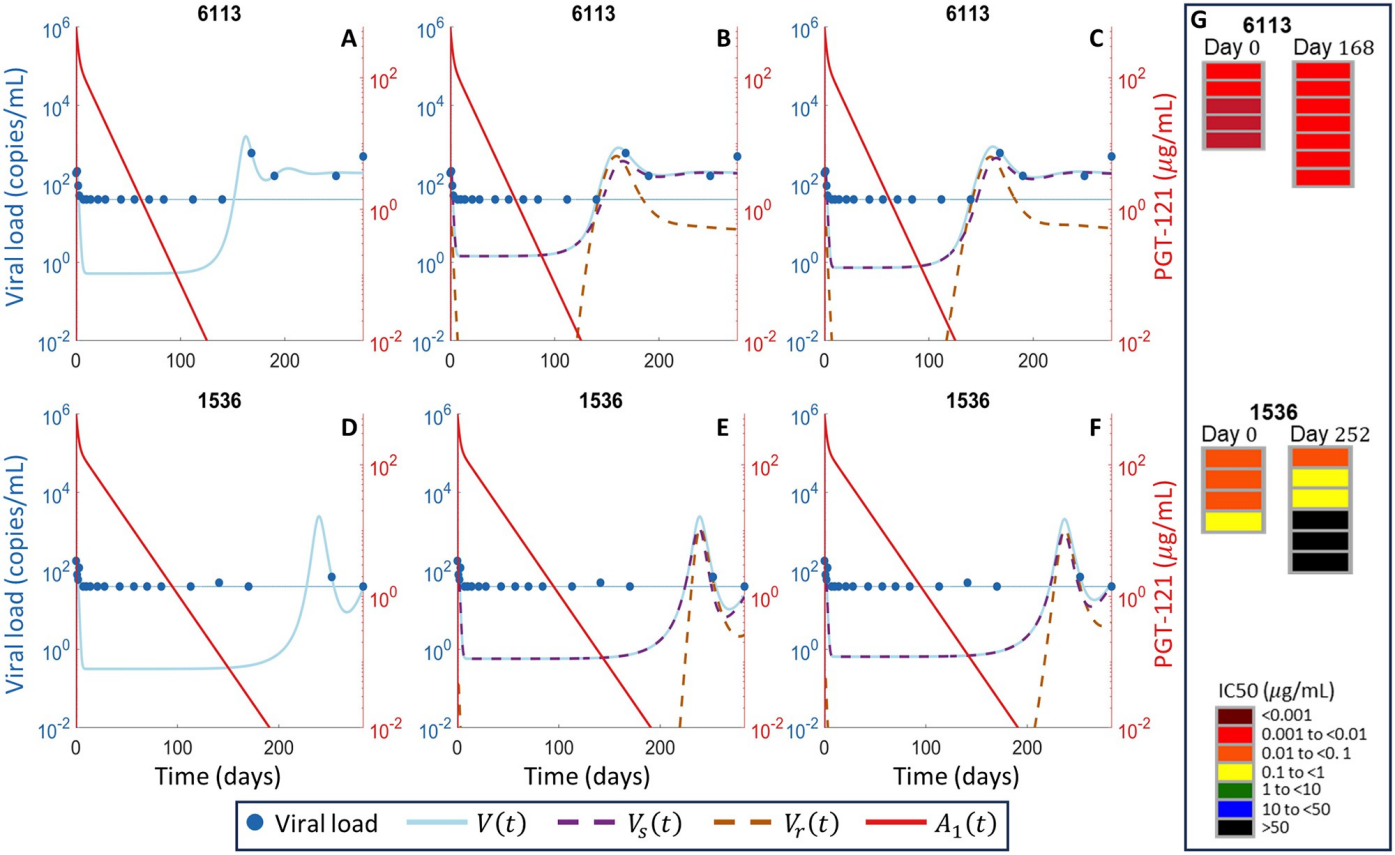
Model fits to long-term controllers. The viral load data from participants 6113 and 1536 is shown in blue circles, the LLoQ is shown as a horizontal dotted blue line, and the simulated total viral load *V*(*t*) is shown in solid blue. The PGT121 resistant population *V*_*r*_(*t*) is shown in dashed orange while the sensitive population *V*_*s*_(*t*) is in dashed purple. The simulated PGT121 concentrations obtained from Eq (1) are shown in solid red. Panels (A) and (D) are the single strain model (Eq (8)), panels (B) and (E) are the two population model without mutation (Eq (9)), and panels (C) and (F) are the two population model with mutation (Eq (10)). The *in vitro* neutralization IC_50_ results, with the time the corresponding samples were taken, from [9] are shown in Panel G.

We fit three mathematical models (Fig 2) to the responder participant data from the PGT121 clinical trial. One model had only a single viral population, whereas the other two models had both PGT121 sensitive and resistant viral subpopulations, with one of these models allowing mutation during PGT121 therapy to generate resistance in addition to baseline resistance, which was assumed to be present in both models as the mean time since HIV diagnosis for study participants was three years. All three models fit the data and we give the best fit parameters each model in Tables A, C, and D of S1 Text for the 8 participants who did not exhibit long-term viral control. We show the fitting results for the three models, Eqs (5)–(7), for the 8 participants who did not exhibit long-term viral control in Figs 3–5. For the participants who exhibited long-term viral control, we used the three mathematical models with latent cell reactivation, given in Eqs (8)–(10). The best-fit model parameters for these long-term controllers are given in Tables E and F of S1 Text. We show the best fits to these long-term participants for all three models in Fig 6.

We used the Bayesian information criterion (BIC) to compare model fits to data. In 6/8 participants who did not exhibit long-term control, including both participants with high viral load and partial baseline sensitivity to PGT121 (PTID: 2319 and 2990), the single viral population model had the lowest BIC (Table 1). However, the single population model is unable to capture the development of resistance to PGT121 that was observed during the PGT121 clinical trial as it does not distinguish between sensitive and resistant viral populations.

**Table 1:** Table of BIC values for individual participants and mathematical models. The BIC value for each participant following fitting using each mathematical model with the lowest BIC value for each participant in bold text. We only fit models with latent cell reactivation to the participants who exhibited sustained viral control (PTID: 6113 and 1536), shown in the lower sub-table. Lower BIC indicates a better model fit [56].

PTID	Single population with reactivation	Two populations without mutation and with reactivation	Two populations with mutation and reactivation
6292	**-21.59**	-19.02	-19.06
6775	-11.86	**-13.21**	-10.90
2990	**-7.09**	-2.39	-4.85
2305	**-21.68**	-9.64	-9.88
7190	**-10.44**	-8.08	-8.33
2319	**-12.01**	-8.50	-10.15
2936	-14.01	-16.98	**-18.47**
4236	**-16.80**	-10.97	-12.34
PTID	Single population	Two populations without mutation	Two populations with mutation
6113	**-40.83**	-34.89	-34.78
1536	**-31.71**	-25.80	-25.61

In fact, *in vitro* neutralization assays indicate that 8/10 trial participants were sensitive to PGT121 at baseline and entirely resistant at viral rebound [9]. This evolution of viral composition and the resulting antibody resistance is captured by both two population viral models given by Eqs (6) and (7). Although these models simplify the numerous pathways to resistance, the two-population viral models, both with and without mutation driven resistance, accurately represented the viral load data. Further, these two models both predicted viral composition at baseline and rebound that were consistent with *in vitro* assays in all cases (Figs 4 and 5). In general, the BIC does not suggest a strong preference for either of the two viral subpopulation models, as equivalent model fits could be obtained without including mutation driven resistance for all participants except 6775 and 2990. The model without mutation (Eq (6)) is preferred for participant 6775, while the model with mutation (Eq (7)) is preferred for participant 2990, as measured by a difference of BIC values greater than 2 [56, 57]. Of the two population models, the lowest BIC was obtained for model with mutation (Eq (7)) in 7/8 participants who did not exhibit long-term control. While the difference in BIC was less than two in 8/10 participants, the BIC suggests a slight preference for the two population model with mutation which is consistent with the emergence of resistance conferring mutations observed in genetic sequencing [9]. This slight preference for the two population model with mutation suggests the role of both the baseline prevalence of the resistant subpopulation, given by *ρ*, and resistance conferring mutations, as pathways to PGT121 resistance. Conversely, the model without mutation has a lower BIC for both long-term controllers, which is consistent with the genetic sequencing of rebound viruses in these participants. Specifically, for participant 1536, the rebound viruses were closely related to the baseline virus [9], while for participant 6113, the rebound viruses maintained sensitivity to PGT121 as shown in Fig 6.

**Fig 7:**
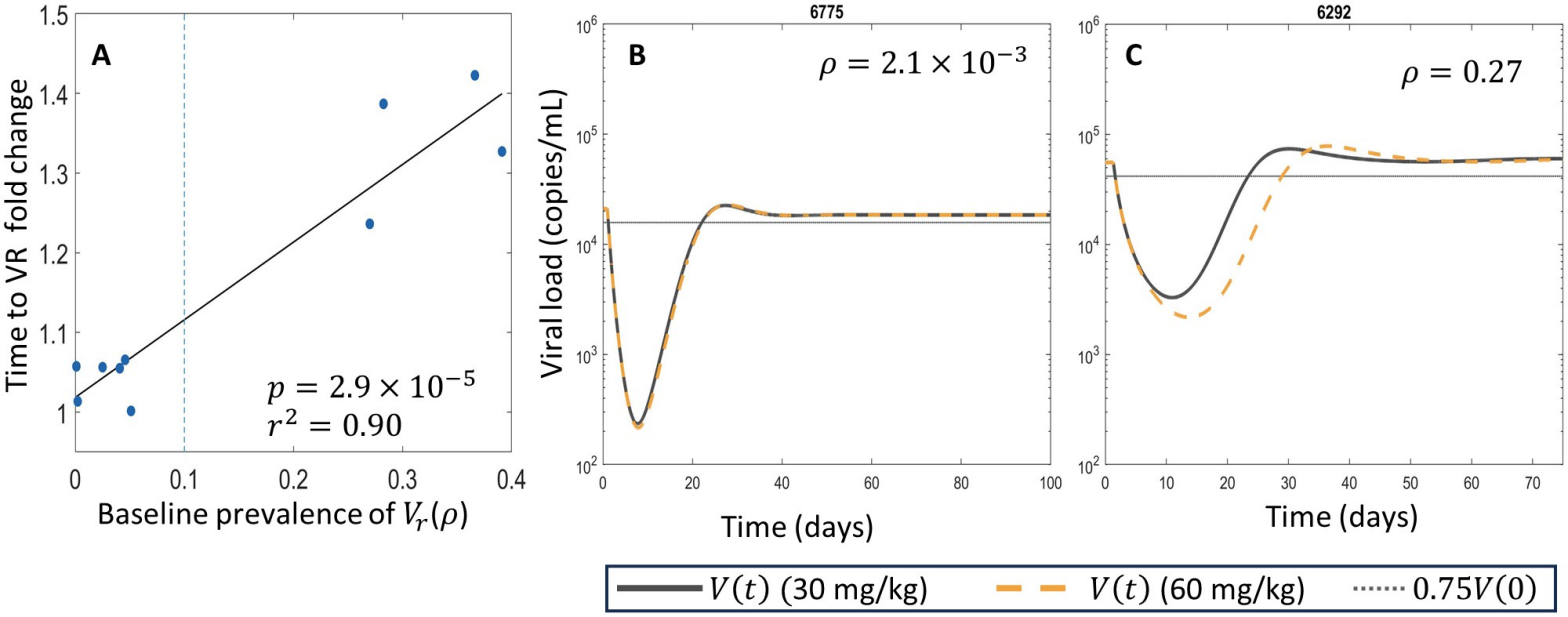
Baseline resistance determines sensitivity to an increased dose of PGT121. Panel A shows a scatter plot of the fold increase in the time to viral rebound (VR) for participants in the simulated trial of 60 mg/kg PGT121 compared to 30 mg/kg PGT121 plotted against the baseline proportion of resistant virus, *ρ* for the two population model with mutation (Eqs (7) and (10)). In panel A, the vertical dotted line at *ρ* = 0.1 distinguishes between the groups of participants with relatively rare (*ρ* < 0.1) and common resistant virus. Panels B and C show the predicted viral load dynamics for participants 6775 and 6292 following administrations of both 30 mg/kg and 60 mg/kg of PGT121 using Eq (7). The fit values of *ρ* are shown for each participant and the corresponding result for the two-population model without mutation, Eq (6), is shown in Fig E of S1 Text.

Including the resistant viral population necessitated the inclusion of two extra parameters, *β*_*r*_ and *α*_*r*_. Despite the improved fit to the measured viral load data, the addition of these two extra parameters decreases the information imparted by the model, and the BIC does not indicate that either of the two population models most efficiently explain the viral load data. The single population model can be considered as a specific case of the two-population models obtained by setting *ρ* = 0 and *μ* = 0. When considering the viral load data alone, the likelihood-ratio test does not support the inclusion of the resistant virus population at the 95% confidence level. This complicates the practical estimation of *ρ*, as the 95% confidence interval for *ρ* thus includes *ρ* = 0. This preference for the single population model, as measured by BIC, is similar to what was observed in [24] and results from the good agreement between all models and the viral load data. However, as previously mentioned, the single population model is unable to capture the PGT121 resistance measured through *in vitro* neutalization assays. Further, our local sensitivity analysis quantifies how sensitive the predicted viral load nadir is to the baseline proportion of resistance, *ρ* and the results in Fig 7 illustrate the sensitivity of viral load dynamics to *ρ* for two dosing strategies.

Our identifiability analysis (see S1 Text) showed that the profile likelihoods have finite confidence intervals for the model parameters *τ*, *α*_*r*_, and *ω*. However, the analysis also showed that *α*_*s*_ was practically non-identifiable. The non-identifiability of *α*_*s*_ can be understood by noting that infection by the sensitive viral population is effectively totally blocked by PGT121, so the rebound dynamics are entirely determined by the resistant virus. Thus, increasing *α*_*s*_ does not change the dynamics of the total viral load. Further, it is well known that the viral production rate *p* is unidentifiable when fitting viral dynamics models to viral load data alone [24, 58]. We discuss the identifiability of the viral production rate, *p*, in the S1 Text.

While we cannot precisely estimate *α*_*s*_, as its 95% confidence estimate is unbounded above, our two-population model fits still identify a clear distinction between the neutralization efficacy of PGT121 against the sensitive and resistant subpopulations. Specifically, we estimate *α*_*s*_ ⩾ 1 for all participants in both the model without and with mutation, see Table C and D of S1 Text. Furthermore, we estimate *α*_*r*_ ≈ 10^−1^ in all participants except the two long-term controllers (Table C and D of S1 Text). These thresholds are qualitatively consistent with the definition of PGT121 resistance used by Stephenson et al. [9], where an IC_50_ > 1, i.e *α*_*r*_ < 1, was used to distinguish resistant viruses from sensitive viruses with IC_50_ < 1, i.e *α*_*s*_ > 1.

Establishing a quantitative relationship between the estimates of *α*_*s*_ and *α*_*r*_ and the *in vitro* neutralization efficacy studies could potentially identify any *in vivo* potency reduction for PGT121, as has been hypothesized [59] and observed in the antibody mediated prevention (AMP) studies [60]. However, identifying such a relationship is complicated by the high neutralization potency of PGT121, where most participants have viruses with IC_50_ below the lowest concentration tested of 0.001*μ*g/mL, Conversely, most resistant viral subspecies have IC_50_s above the highest concentration tested in *in vitro* neutralization assays. Further, our estimates for *α*_*s*_ and *α*_*r*_ represent the neutralization sensitivity of the sensitive and resistant subpopulations, rather than for one individual viral strain.

### Baseline PGT121 resistance determines relative viral fitness

The infection rate constants are calculated in Eq (12) and explicitly link the subpopulation specific infection rates, *β*_*s*_ and *β*_*r*_, to the baseline concentration of sensitive and resistant viral subpopulations, *V*_*s*_(0) and *V*_*r*_(0), respectively. Specifically, using the expressions for *β*_*s*_ and *β*_*r*_, in Eq (12), and the expressions for *I*_*s*_(0) and *I*_*r*_(0) in Eq (11), we immediately see

$$\begin{array}{cc} \frac{\beta_{r}}{\beta_{s}} & =(\frac{\delta {\left[I_{r}\left(0\right)\right]}^{\omega }/\left[fT\left(0\right)V_{r}\left(0\right)\right]}{\delta {\left[I_{s}\left(0\right)\right]}^{\omega }/\left[fT\left(0\right)V_{s}\left(0\right)\right]})={\left(\frac{\rho }{1-\rho }\right)}^{\omega -1} \end{array}$$

which yields an explicit link between the baseline level of PGT121 resistance, *ρ*, and the relative fitness of the sensitive and resistant subpopulations. Specifically, we note that as the resistant subpopulation becomes increasingly rare, characterised by *ρ* → 0, the resistant subpopulations has lower relative fitness in the absence of PGT121 treatment, with *β*_*r*_/*β*_*s*_ → 0.

Similarly, if the majority of the virus is sensitive to PGT121 at baseline, so *V*_*r*_(0)<*V*_*s*_(0), then *β*_*r*_ < *β*_*s*_. As the remaining parameters are conserved between the viral populations *V*_*s*_ and *V*_*r*_, the cost of resistance to PGT121 is completely encoded in the infection rate *β*_*r*_ relative to that of the sensitive virus *β*_*s*_.

Finally, the sensitive and resistant populations are competing to infect the same population of target cells. Thus, in the absence of therapy, the higher infectivity rate, *β*_*s*_, ensures that the sensitive viral subpopulation will out-compete the resistant population. Indeed, our model simulations indicate eventual re-sensitization to PGT121 when extrapolating over a longer duration than the PGT121 clinical trial.

### Relative viral fitness determines dynamical pathways to resistance

Our modeling identifies the role of the relative fitness of PGT121 sensitive and resistant strains in the dynamical pathway to resistance and viral rebound. In one group of participants, our modeling indicates the existence of a rare resistant subpopulation at baseline, which we define by *ρ* < 0.1, so the majority of virus is initially very sensitive to PGT121 (Tables C and D of S1 Text). In this group, the concentration of the rare resistant virus remains approximately constant at the beginning of therapy and only begins to rise after the sensitive virus has fallen. The resistant subpopulation of virus then expands to comprise almost the entire viral population during rebound (cf. participant 6775 in Figs 4 and 5). In a second group, the baseline virus has a relatively frequent resistant viral subpopulation. This resistant subpopulation is still slightly sensitive to PGT121 and falls while the PGT121 concentration remain high. Eventually, the PGT121 concentrations fall low enough to allow for the resistant subpopulation to drive viral rebound while still suppressing the sensitive viral subpopulation (cf. participant 6292 in Figs 4 and 5).

These distinct pathways to the development of resistance are potentially clinically relevant. For example, in the participants where the resistant subpopulation is relatively frequent but slightly sensitive to PGT121, increasing the dose could drive deeper decay of the resistant virus and thus delay viral rebound. Conversely, in the remaining participants where a single administration of PGT121 does not drive decay of the rare resistant subpopulation, increasing the dose would be unlikely to postpone the time to viral rebound. To test this hypothesis, we simulated a theoretical trial of 60 mg/kg PGT121 in the identical trial population, which is double the dose administered in the phase I trial of PGT121 [9]. We defined the time to viral rebound as the first time the viral load reaches 75% of the baseline viral load post-treatment. We computed the fold increase in the time to viral rebound in the simulated trial relative to the Phase I trial data. As expected, increasing the amount of PGT121 administered increased the time until viral rebound when using both the two population model without mutation (in Eq (6)), and with mutation (in Eq (7)). However, with both models, there is a clear distinction between the participants with the rare resistant subpopulation at baseline and the remaining participants. We show a scatter plot of the relative increase in time to viral rebound against the proportion of resistant virus at baseline in Fig 7. We also show the predicted viral dynamics for participants 6775 and 6292 as representative participants from either group. In participant 6775, who has rare baseline resistance, the simulated viral response to 30 and 60 mg/kg of PGT121 is nearly identical. Conversely, doubling the dose of PGT121 increases the predicted time to viral rebound of participant 6292 by 6 days, illustrating the differences between the two groups of participants. We identified a significant positive correlation between the baseline resistance, *ρ*, and the relative change in the time to viral rebound shown in Fig 7.

Three of the four participants in the low viral load group (participants 6113, 1536, and 2936) as well as participants 6775 and 7190 are predicted to have a relatively rare resistant subpopulation that comprises less than 10% of the baseline virus load for both the two population models with (Eq (7)) and without mutation (Eq (6)). For the remaining participant in the low viral load group (4236), the two population model with mutation predicts that the resistant subpopulation is rare at baseline, which is consistent with the *in vitro* neutralization results of [9].

In the participants who do not exhibit long term control, the viral load rebounds rapidly to baseline levels (cf. Figs 4 and 5). In these participants, the resistant virus expands as the sensitive virus decays and thus drives viral rebound. This modeling prediction is consistent with the *in vitro* neutralization studies that measured extremely sensitive virus at baseline and completely resistant virus at rebound [9] shown in Figs 4 and 5. These neutralization assays measure the neutralization IC_50_ for distinct pseudovirus strains sampled pre- and post-PGT121 treatment. While our models do not allow for a one-to-one comparison of viral composition with the *in vitro* assays, the dominance of the resistant subpopulation upon rebound in our model simulation is consistent with the *in vitro* neutralization data.

Our model predicts the presence of baseline resistant subpopulations that comprise between 10% and 40% of baseline virus (participants 6292, 2305, and 4236 in the two population model without mutation, Eq (6), and participants 6292, 2990, 2305, and 2319 in the two population model with mutation, Eq (7)). For the partially sensitive participants, 2990 and 2319, the *in vitro* neutralization assays showed preexisting resistance to PGT121 in the initial viral sample [9]. In the remaining participants with *ρ* > 0.1, our modeling predicts the presence of a resistant viral subpopulation that was not identified during the *in vitro* neutralization assays [9]. In a related modeling study of the bnAbs 3BNC117 and 10–1074, Meijers et al. [61] also predicted higher proportions of resistant virus at baseline that predicted from *in vitro* neutralization assays. These *in vitro* assays only measure the sensitivity of pseudo-viruses made from virus that were circulating in sufficiently large concentrations to be sampled. Stephenson et al. concluded that the multiple genetic pathways to resistance observed in all the trial participants suggests that these resistant viruses were indeed present at baseline [9]. In all these participants, our model predicts that viral population will be entirely resistant upon viral rebound, consistent with the post-treatment *in vitro* assay.

### PGT121 treatment releases competitive suppression of the resistant subpopulation

The resistant subpopulation of virus drives viral rebound and is predicted to compose the majority of rebound virus in participants not exhibiting long-term control. This modeling prediction, consistent with *in vitro* neutralization studies, demonstrates the role of resistance in the loss of effectiveness of PGT121. In our models, the sensitive and resistant viral subpopulations are competing to infect the same population of target cells and the cost of resistance [62] is encoded by the infection rates *β*_*r*_ < *β*_*s*_. Thus, in the absence of therapy, the higher infectivity rate *β*_*s*_ ensures that the sensitive viral subpopulation competitively suppresses–but does not exclude– the resistant viral population.

However, PGT121 therapy imposes a selection pressure on the sensitive subpopulation and confers a treatment-mediated relative fitness advantage to the resistant viral subpopulation. The temporal dynamics of the resistant viral population demonstrates that the selection pressure imposed by PGT121 against the sensitive virus decreases it’s population size and therefore the competitive suppression of the resistant population which then drives the resulting viral rebound. This treatment mediated release of competitive suppression has been observed in bacteria [63], malaria [64, 65], and cancer [66, 67]. In participant 6292, our modeling predicts that, as the PGT121 concentration falls, the resistant virus is being replaced by the sensitive virus due to competition by day 56 post treatment leading to eventual re-sensitization, see Figs 4 and 5, and our modeling predicts the sensitive viral subpopulation will be dominant within 16 days after the final data point.

### Re-sensitization to PGT121

In both two viral population models (Eqs (6) and (7)), our modeling predicts that the sensitive strain will eventually out-compete the resistant strain as the selection pressure imposed by PGT121 is eased during antibody washout, although this re-sensitization is predicted to occur after the final data point in all participants except 6113 and 1536. We define re-sensitization as the first time following viral rebound where the sensitive viral population is more common than the resistant viral population. Our modeling predicts that the median time lag between viral rebound and re-sensitization is 122 and 210 days for the models in Eq (6) and Eq (7), respectively. Further, the median time from treatment to re-sensitization is 150 and 238 days for models Eq (6) and Eq (7), respectively.

To understand the mechanisms underlying this viral replacement and re-sensitization, we noted that our fitting predicts a large range of baseline sensitivity to PGT121 with *α*_*s*_ spanning 4 orders of magnitude. We identified a positive correlation between log(*α*_*s*_) and the time to re-sensitization in both models, Eq (6) and Eq (7) (*p* = 0.003, *r* = 0.79 and *p* = 0.007, *r* = 0.74, respectively). We show the corresponding scatter plots in Fig G of S1 Text. Despite this correlation, our local sensitivity analysis (see Methods) indicates that changes in the baseline sensitivity to PGT121 are only responsible for a small change in the time from treatment to re-sensitization. As PGT121 decays exponentially during the elimination phase, a 10% change in PGT121 sensitivity, i.e, *α*_*s*_, only translates to an approximately two day difference in the time for PGT121 concentrations to reach the half-effect concentration. Rather, a simple analysis, given in S1 Text, suggests that the time required for PGT121 concentrations to decay to the half-effect concentration is responsible for the resulting change in time to re-sensitization. This further supports the conclusion that the time to re-sensitization is determined by the time lag between PGT121 administration and the corresponding removal of the treatment-mediated selection pressure on the sensitive virus. Prior to re-sensitization, the circulating virus is dominated by the resistant subpopulation as the sensitive virus is controlled by the circulating PGT121. Consequently, additional doses of PGT121 would deepen the suppression of the sensitive virus without necessarily decreasing the circulating viral load due to the dominance of the resistance subpopulation.

### High potency of PGT121 supports long-term viral control

In both long-term viral controllers, participants 6113 and 1539, our model fit suggests viral rebound is driven by the emergence of the resistant viral population, shown in Fig 6. However, our model predicts that, in contrast to the other participants, the virus in the long-term controllers is rapidly re-sensitized to PGT121 following rebound. Along these lines, the long-term controllers retained sensitivity to PGT121 long after administration of PGT121 and viral rebound occurs long after PGT121 has become undetectable, despite being driven by the resistant virus. These modeling predictions are consistent with the *in vitro* neutralization assays that show the rebound virus retains at least partial sensitivity to PGT121 in the two long-term controllers [9] shown in panel G of Fig 6. In particular, both our modeling and the *in vitro* neutralization assay indicate that the rebound virus, sampled on day 256, in participant 1536 is partially sensitive to PGT121. Specifically, the PGT121 sensitive subpopulation comprises 32% of the rebound virus in our model without mutation Eq (9) and 43% in the model with mutation Eq (10).

The model without mutation, Eq (9), predicts that the sensitive subpopulation represents a slight majority, 57%, of the viral load at day 168 for participant 6113 while the model with mutation, Eq (10), predicts that 62% of the viral load is sensitive to PGT121. The *in vitro* neutralization assay of virus sequenced on day 168 from participant 6113 indicates full sensitivity to PGT121, but this is based on analysis of only 7 psuedo-virus strains.

Finally, as we included latent cell reactivation in the model, the model predicts low-level viremia below the level of detection throughout the period of viral control (Fig 6). As our model captures the viral dynamics without the inclusion of a cellular immune response that is included in other models [13, 39], we would expect viral rebound to rapidly follow PGT121 washout. However, the sustained viral control despite sustained low-level viremia due to latent reactivation suggests that the high neutralization potency of PGT121 at low concentrations is responsible for the viral control exhibited by participants 6113 and 1539.

## Discussion

PGT121 has shown promise in preclinical trials to treat HIV-1 infections in humans, and a recent phase I clinical trial demonstrated the potent neutralization effect of PGT121 in HIV-1 positive participants. However, as has been the case with most existing bnAbs [68–70], resistance to PGT121 limits the clinical applicability of this antibody when used as monotherapy.

In this work, we used mathematical modeling to explore the mechanisms underlying this development of resistance in participants with chronic viremia in a recent phase I clinical trial. We adapted a viral dynamic model to include the effect of PGT121 through a delayed neutralization term and the inclusion of both sensitive and resistant subpopulations of virus as in prior work [24]. We then fit the mathematical model to plasma viral load data for each participant and estimated the sensitivity of the sensitive and resistant viral subpopulations to PGT121. Our modeling indicated the importance of the relative fitness of sensitive and resistant subpopulations to PGT121 in driving viral rebound.

In particular, our modeling predicts that some participants have relatively common PGT121 resistant virus at baseline, which may have been nevertheless not been detected in the baseline virus sample. We also identified a group other participants with rare pre-existing resistant virus, which is consistent with *in vitro* neutralization assays of virus isolated from participants in this trial. All told, our results suggested that pre-existing resistance is an important mechanism underlying viral rebound and treatment resistance. While 8/10 of the participants were classified as entirely sensitive to PGT121 at baseline, the distinct genetic pathways to resistance are suggestive of pre-existing PGT121 resistance [9]. Our conclusion is consistent with other recent modeling studies of bnAb. For example, when considering a clinical trial of VRC01 treatment administered post ART interruption, Saha and Dixit [71] identified the role of pre-existing resistant virus to VRC01 in the latent reservoir in driving viral rebound. Reeves et al. [59] emphasized the resistant fraction of founder virus as a key component leading to the failure of AMP trials. These complementary studies demonstrate the role of pre-existing resistance in determining bnAb treatment efficacy across different stages of HIV-1 infection.

In the eight participants with viral rebound occurring within 28 days post treatment, our model indicates that viral rebound is due to the decline of competitive suppression of the resistant virus as the PGT121 concentration falls. In these participants, the high neutralization potency of PGT121 imposes severe selection pressure on the sensitive virus. This selection pressure provides a fitness advantage to the resistant virus, which leads to rapid expansion of the resistant subpopulation and viral rebound. In these participants, the resistant subpopulation is not very sensitive to PGT121 and is able to exploit the treatment mediated fitness advantage. The selection of resistant viruses has been observed in clinical studies of other bnAbs [3–5, 62]. As would be expected, sensitivity analysis of model parameters showed that increasing the sensitivity of the resistant virus to PGT121 delayed viral rebound and decreased the viral nadir. Our modeling also predicts that, as the selection pressure imposed by PGT121 is eased due to antibody washout, the sensitive virus will once again out-compete the resistant virus leading to re-sensitization of the total viral population. This replacement and subsequent re-sensitization has been observed experimentally [3]. Finally, we also simulated a theoretical trial of a dose of 60 mg/kg PGT121. The baseline prevalence of the resistant subpopulation determined the relative change in the time to viral rebound in this theoretical trial compared with the phase I trial of PGT121. Interestingly, we predict that participants with a rare resistant subpopulation would not benefit from an increased dose of PGT121. Taken together, our modeling suggests that the characteristics of a possibly rare but pre-existing resistant subpopulation, as well as resistance generated by mutation, determine viral dynamics following PGT121 treatment, and further support the need for combination bnAb therapies [12, 22, 72]. Indeed, a bnAb cocktail containing PGT121 and a complementary bnAb, PGDM1400, protected rhesus macaques from a mixed SHIV challenge while both PGT121 and PGDM1400 alone failed to protect against SHIV infection [12]. Similarly, a single administration of a triple bnAb cocktail of PGT121, PGDM1400, and VRC07–523LS given to PWLH reduced circulating HIV-1 levels by a mean of 2 log_10_ copies/mL but rebound occurred within a median of 20 days post viral load nadir with rebound viruses displaying partial or complete resistance to both PGT121 and PGDM1400 [23].

We used our mathematical model to explore the mechanism behind the long-term viral control post treatment in participants 6113 and 1536. Our results indicated that the observed long-term viral control may be due to the high neutralization potency of PGT121, and that even with low-level sustained viremia due to latently infected cell reactivation, levels of PGT121 below the limit of detection were able to inhibit viral rebound for a few months. Previous studies in both humans and rhesus macaques have suggested that bnAb therapy can enhance HIV specific T-cell responses [73, 74]. Stephenson et al. [9] thus looked for any enhancement in the HIV T-cell response in both long-term controllers. They found no increase in the number of HIV-1 specific T-cells nor an increase in the breadth of T-cell response, supporting the conclusion that the long-term viral control was due to the neutralization potency of PGT121 alone. As measurements of PGT121 concentrations in tissue are not available, it is possible that such concentrations remained elevated despite circulating PGT121 concentrations falling below the limit of detection during the observed long-term viral control. Once again, our modeling indicates that the resistant subpopulation causes viral rebound, but is rapidly replaced by the sensitive virus in these two subjects as the neutralizing effect of PGT121 wanes. Moreover, our parametrization suggests that the resistant strain in the long-term controllers is significantly more sensitive to PGT121 than the resistant strains in the other eight participants. These results indicate that long-term viral suppression is possible following PGT121 therapy, but that this suppression is predicated on the existence of relatively sensitive pre-existing resistant viral subpopulations and the resulting long-term neutralization effect of PGT121.

Our modeling has some limitations. We made the simplifying assumption that interaction between PGT121 and the virus did not impact the clearance rate of the antibody. However, *in vivo* data suggests that PGT121 has a shorter half-life in HIV positive individuals than in uninfected individuals [9] potentially due to clearance of antibody-virus complexes. Omitting this effect is a possible limitation of our study. Moreover, we simplified the reactivation process of latently infected cells and discounted the temporal dynamics of the latent reservoir in our modeling of both long-term controllers. Further, our modeling drastically oversimplifies the viral diversity present in participants by assuming that virus is either resistant or sensitive to antibody treatment. These assumptions simplify the underlying biological mechanisms and they allow us to study the broad impact of each mechanism without overcomplicating the mathematical models. However, it is important to note that fitting the viral load data alone does not indicate a preference for models that include a viral subpopulation resistant to PGT121. Despite the improved model fits as measured by log-likelihood, the inclusion of two extra model parameters in our two viral population models imparts less information, as measured by BIC, than the one population model. Nevertheless, our two population models qualitatively predict the results of the *in vitro* neutralization assays that clearly identify the emergence of resistance to PGT121. Finally, while our modeling accurately predicts the results of the *in vitro* neutralization assays taken shortly after viral rebound, we were not able to compare our re-sensitization predictions against *in vitro* neutralization data, although re-sensitization has been experimentally observed in a study using the bnAb 3BNC117 [3].

In summary, we have developed and tested three mathematical models to explore the development of resistance to the bnAb PGT121. We fit these models to participant data, and tested our model predictions against the qualitative results of *in vitro* neutralization assays. Our modeling suggests that competitive release of a resistant virus population may drive viral rebound, although this resistance may not be permanent. Our models indicate that, if the expansion of this resistant population can be avoided through combination therapies, then highly potent bnAbs such as PGT121 may be a promising components of therapeutic interventions to induce long-term viral suppression in HIV-1 positive individuals.

## Supporting information

S1 Text Supporting information: Modeling resistance to the broadly neutralizing antibody PGT121 in people living with HIV-1. (PDF)
