# Supplementary material for: Modeling resistance to the broadly neutralizing antibody PGT121 in people living with HIV-1
Source: PLoS Comput Biol. 2024 Mar 29;20(3):e1011518. doi: 10.1371/journal.pcbi.1011518 (PMC11006161; doi:10.1371/journal.pcbi.1011518)
Supplement: S1 Text — (PDF) [file pcbi.1011518.s001.pdf]

S1 Text: Modeling resistance to the broadly neutralizing antibody PGT121 in people living with HIV-1

Tyler Cassidy[1], Kathryn E. Stephenson[2,3,4], Dan H. Barouch[2,3,4], Alan S. Perelson[5,*]

1 School of Mathematics, University of Leeds, Leeds, United Kingdom

2 Center for Virology and Vaccine Research, Beth Israel Deaconess Medical Center, Boston, Massachusetts, United States of America

3 Division of Infectious Diseases, Beth Israel Deaconess Medical Center, Boston, Massachusetts, United States of America

4 Ragon Institute of MGH, MIT and Harvard, Cambridge, Massachusetts, United States of America

5 Theoretical Biology and Biophysics, Los Alamos National Laboratory, Los Alamos, New Mexico, United States of America

We present the local sensitivity and identifiability analysis for the viral dynamics models given in the main text. For ease of presentation, we restate the models here. The single population viral dynamics model is given by

$$\left.\begin{aligned} \frac{\mathrm{d}}{\mathrm{dt}}T(t) &= \lambda - \hat{\beta}(t)T(t)V(t) - dT(t) \\ \frac{\mathrm{d}}{\mathrm{dt}}I(t) &= f\hat{\beta}(t)T(t)V(t) - \delta[I(t)]^{\omega} \\ \frac{\mathrm{d}}{\mathrm{dt}}V(t) &= pI(t) - cV(t) \end{aligned}\right\} \tag{S1}$$

with model terms given in the main text. The two population models without, and with, mutation are respectively given by

$$\left.\begin{aligned} \frac{\mathrm{d}}{\mathrm{dt}}T(t) &= \lambda - \hat{\beta}_s(t)T(t)V_s(t) - \hat{\beta}_r(t)T(t)V_r(t) - dT(t) \\ \frac{\mathrm{d}}{\mathrm{dt}}I_s(t) &= f\hat{\beta}_s(t)T(t)V_s(t) - \delta[I_s(t)]^{\omega} \\ \frac{\mathrm{d}}{\mathrm{dt}}V_s(t) &= pI_s(t) - cV_s(t) \\ \frac{\mathrm{d}}{\mathrm{dt}}I_r(t) &= f\hat{\beta}_r(t)T(t)V_r(t) - \delta[I_r(t)]^{\omega} \\ \frac{\mathrm{d}}{\mathrm{dt}}V_r(t) &= pI_r(t) - cV_r(t) \end{aligned}\right\} \tag{S2}$$

and

$$\left.\begin{aligned}
\frac{\mathrm{d}}{\mathrm{dt}}T(t) &= \lambda - \hat{\beta}_s(t)T(t)V_s(t) - \hat{\beta}_r(t)T(t)V_r(t) - dT(t) \\
\frac{\mathrm{d}}{\mathrm{dt}}I_s(t) &= f(1-\mu)\hat{\beta}_s(t)T(t)V_s(t) - \delta[I_s(t)]^{\omega} \\
\frac{\mathrm{d}}{\mathrm{dt}}V_s(t) &= pI_s(t) - cV_s(t) \\
\frac{\mathrm{d}}{\mathrm{dt}}I_r(t) &= f\hat{\beta}_r(t)T(t)V_r(t) + f\mu\hat{\beta}_s(t)T(t)V_s(t) - \delta[I_r(t)]^{\omega} \\
\frac{\mathrm{d}}{\mathrm{dt}}V_r(t) &= pI_r(t) - cV_r(t).
\end{aligned}\right\} \tag{S3}$$

The single population model with latent cell reactivation is given by

$$\left.\begin{aligned}
\frac{\mathrm{d}}{\mathrm{dt}}T(t) &= \lambda - \hat{\beta}T(t)V(t) - dT(t) \\
\frac{\mathrm{d}}{\mathrm{dt}}I(t) &= aL_0 + f\hat{\beta}(t)T(t)V(t) - \delta[I(t)]^{\omega} \\
\frac{\mathrm{d}}{\mathrm{dt}}V(t) &= pI(t) - cV(t).
\end{aligned}\right\} \tag{S4}$$

Finally, the two population models that include latent cell reactivation without, and with, mutation respectively are given by

$$\left.\begin{aligned}
\frac{\mathrm{d}}{\mathrm{dt}}T(t) &= \lambda - \hat{\beta}_s(t)T(t)V_s(t) - \hat{\beta}_r(t)T(t)V_r(t) - dT(t) \\
\frac{\mathrm{d}}{\mathrm{dt}}I_s(t) &= aL_0 + f\hat{\beta}_s(t)T(t)V_s(t) - \delta[I_s(t)]^{\omega} \\
\frac{\mathrm{d}}{\mathrm{dt}}V_s(t) &= pI_s(t) - cV_s(t) \\
\frac{\mathrm{d}}{\mathrm{dt}}I_r(t) &= f\hat{\beta}_r(t)T(t)V_r(t) - \delta[I_r(t)]^{\omega} \\
\frac{\mathrm{d}}{\mathrm{dt}}V_r(t) &= pI_r(t) - cV_r(t)
\end{aligned}\right\} \tag{S5}$$

and

$$\left.\begin{aligned}
\frac{\mathrm{d}}{\mathrm{dt}}T(t) &= \lambda - \hat{\beta}_s(t)T(t)V_s(t) - \hat{\beta}_r(t)T(t)V_r(t) - dT(t) \\
\frac{\mathrm{d}}{\mathrm{dt}}I_s(t) &= aL_0 + f(1-\mu)\hat{\beta}_s(t)T(t)V_s(t) - \delta[I_s(t)]^{\omega} \\
\frac{\mathrm{d}}{\mathrm{dt}}V_s(t) &= pI_s(t) - cV_s(t) \\
\frac{\mathrm{d}}{\mathrm{dt}}I_r(t) &= f\hat{\beta}_r(t)T(t)V_r(t) + f\mu\hat{\beta}_s(t)T(t)V_s(t) - \delta[I_r(t)]^{\omega} \\
\frac{\mathrm{d}}{\mathrm{dt}}V_r(t) &= pI_r(t) - cV_r(t).
\end{aligned}\right\} \tag{S6}$$

Latently infected cell reactivation rate

Including reactivation from the latent reservoir during long-term viral control only requires estimating one extra parameter, the product aL_0. However, there is little information available on the size of the latent reservoir or the

reactivation rate in the study participants. Therefore, we use information from the viral load data to derive a maximal value of the product aL_0. Then, for different fixed values of aL_0, we re-fit the model to the data and estimated the remaining model parameters. To determine a upper bound for aL_0, we estimate

$$\frac{\mathrm{d}}{\mathrm{dt}} I(t) \geqslant aL_0 - \delta I^{\omega},$$

which would correspond to no productive infection during treatment. Then, if the concentration of productively infected cells reaches a treated equilibrium value I^* during PGT121 induced viral control, we have

$$I^* \geqslant \left(\frac{aL_0}{\delta}\right)^{1/\omega}.$$

Using this steady state estimate for I^*, the corresponding treated viral load concentration V^* is at least

$$V^* \geqslant \frac{p}{c}\left(\frac{aL_0}{\delta}\right)^{1/\omega},$$

which can be re-arranged to give

$$aL_0 \leqslant \left(\frac{V^* c}{p}\right)^{\omega} \delta.$$

While we do not know the true value of V^*, the HIV assays used in the PGT121 trial had a lower limit of quantification of 40 copies/mL. Thus, for the two controller participants, we bound $V^* \leqslant 40$ copies/mL to calculate an upper bound on the reactivation rate given by

$$aL_0 \leqslant \left(\frac{40c}{p}\right)^{\omega} \delta.$$

By obtaining a first estimate for the parameters p and ω from the viral decay data following administration of PGT121 in participants 1536 and 6113, and then calculating δ through the pretreatment infected steady state assumption detailed earlier, we obtain the upper bound for aL_0. In general, the upper bound for aL_0 was the same order of magnitude as the estimates in Conway and Perelson [1] and is roughly 10-fold larger than the estimates from rhesus macques that received ART during the acute phase of SIV infection [2]. This 10-fold difference from rhesus macaque data may be due to a larger latent reservoir in chronically infected individuals.

Then, we take 20 equally spaced values of aL_0 satisfying

$$aL_0 \in \left[10^{-2} \times \left(\frac{40c}{p}\right)^{\omega} \delta, \left(\frac{40c}{p}\right)^{\omega} \delta\right],$$

and fit equations (S4) and (S5) to the controller data for each value of aL_0.

Model selection

To compare the predictive power of each mathematical model, we compute the Bayesian Information Criteria (BIC) for the i-th participant and j-th model by

$$\mathrm{BIC}_i(\mathrm{Model}_j) = n\log\left(\frac{F_i(\theta_i^*)}{n}\right) + M\log(n)$$

where F_i is given by Eq 12 in the Main Text, M is the number of parameters fit in each model, and n is the number of data points. We list the BIC for each participant and model in Table S1. To compare different models for each participant, we compute

$$\Delta_{jk}\mathrm{BIC}_i = \mathrm{BIC}_i(\mathrm{Model}_j) - \mathrm{BIC}_i(\mathrm{Model}_k)$$

for $j \neq k$. For the i-th participant, we conclude that the BIC indicates that there is evidence against the jth mathematical model in favour of the kth model if $\Delta_{jk}\mathrm{BIC}_i > 2$ [3].

Local sensitivity analysis

We present the results of the local sensitivity analysis in Figures B and C. In all models, the parameters responsible for the effectiveness of the PGT121 (α_s and α_r in (S2) and (S3)) has a notable effect on the viral nadir and time to virus rebound. However, the biological interpretation of this impact differs between the single and two subpopulation models.

Single viral population model

In the single population model, it is to be expected that increasing the neutralization potency of PGT121 via the parameter α decreases the viral nadir and increases the time to virus rebound. However, the effect of variation in ω is less simple to translate into biological mechanisms. We recall that ω acts as an indicator of immune killing of infected cells and note that the viral nadir corresponds to the minimum infected cell concentration. Then, if the infected cell concentration falls below one cells/mL, i.e., $I(t) < 1$, during treatment, $\delta I(t)^{0.9\omega} > \delta I(t)^{\omega} > \delta I(t)^{1.1\omega}$. It therefore follows that

$$\frac{\beta}{1+\alpha A(t)}V(t)T(t) - \delta I(t)^{0.9\omega} < \frac{\beta}{1+\alpha A(t)}V(t)T(t) - \delta I(t)^{\omega} < \frac{\beta}{1+\alpha A(t)}V(t)T(t) - \delta I(t)^{1.1\omega},$$

and, as the viral load rapidly equilibrates with the concentration of infected cells, it is clear why decreasing ω leads to a decrease in viral nadir. Biologically, a paucity of infected cells leads to decreased immune recruitment and correspondingly the immune response, which translates to a larger viral load at nadir. Conversely, infected cells are much more common during viral rebound, so $I(t) > 1$ and

$$\frac{\beta}{1+\alpha A(t)}V(t)T(t) - \delta I(t)^{0.9\omega} > \frac{\beta}{1+\alpha A(t)}V(t)T(t) - \delta I(t)^{\omega} > \frac{\beta}{1+\alpha A(t)}V(t)T(t) - \delta I(t)^{1.1\omega}.$$

The resulting immune recruitment and increased immune effect acts to drive clearance of infected cells, which in turn delays viral rebound, as would be expected.

Two viral population model

In the two population model, changing the sensitivity of the sensitive strain, α_s does not impact the viral nadir concentration nor the time to rebound. As the two subpopulation models include a viral population that is less susceptible to neutralization by the bnAb that drives viral rebound, we hypothesize that increasing the effect of therapy on the sensitive virus is counterbalanced by the hastened competitive release of the resistant population. Interestingly, the small effect of changes in α_s on the time to re-sensitization can be understood from the PGT121 pharmacokinetics. During the elimination phase, the concentration of PGT121 is approximately given by

$$A_1(t) = Ke^{-\lambda t},$$

where K is the plasma concentration of PGT121 following the rapid distribution to the tissues and λ is the slope of the elimination phase on the log scale. For a terminal elimination half life of $t_{1/2}$, λ is given by

$$\lambda = \frac{\log(2)}{t_{1/2}}.$$

Now, $A_1(t)$ reaches the half effect concentration $c_0 = 1/\alpha_s$ at time

$$t_0 = \frac{1}{\lambda}\left[\log(K) - \log(c_0)\right]$$

A 10% increase or decrease in α_s corresponds to decreasing or increasing c_0 to half effect concentrations

$$c_1 = \frac{10}{11}c_0 \quad \text{or} \quad c_2 = \frac{10}{9}c_0$$

respectively. Then, $A_1(t)$ reaches the half effect concentrations $c_{1,2}$ at times $t_{1,2}$ given by

$$t_1 = \frac{t_{1/2}}{\log(2)}\left[\log(K) - \log(c_0) - \log(10/11)\right] \quad \text{and} \quad t_2 = \frac{t_{1/2}}{\log(2)}\left[\log(K) - \log(c_0) - \log(10/9)\right].$$

It follows that

$$t_1 - t_0 = \frac{-t_{1/2}}{\log(2)}\log(10/11), \quad \text{and} \quad t_0 - t_2 = \frac{t_{1/2}}{\log(2)}\log(10/9).$$

Using the estimated PGT121 half life of 13–19 days, we have $t_1 - t_0 \approx$ 1.78–2.61 days and $t_0 - t_2 \approx$ 1.97–2.88 days. Now, the median time to re-sensitization of the two strain model with mutation are 149 and 151 days for the lower and upper values of α_s, respectively. Thus, the approximately 1% change in the time to re-sensitization during local sensitivity analysis can be explained by the change in the time required for PGT121 to decay to the half effect concentrations $c_{1,2}$.

Identifiability analysis

We show representative profile likelihoods in Figure D. For participant 7190, we note that α_r does not have a finite lower bound except for the physiological bound of $\alpha_r > 0$. Further, α_s is not practically identifiable and the 95% confidence interval is unbounded above. This corresponds to increasing neutralization effect of PGT121 on the sensitive population not changing the total population dynamics. The *in vitro* neutralization assays for this participant indicate that the sensitive virus has $\text{IC}_{50} < 0.023\mu$g/mL, which is extremely sensitive, and indicates that PGT121 acts to effectively block all infection by the sensitive strain during viral decay. As viral rebound is driven by the resistant strain, increasing the sensitivity of the sensitive strain will not impact the observed viral rebound dynamics and it is unsurprising that α_S does not have a practically identifiable upper bound.

Profile likelihoods are calculated by fixing a parameter $\theta_{j,i}$ and re-fitting the remaining free parameters to characterise the role of $\theta_{j,i}$ in the likelihood function. In our parameter fitting, the two parameters p and ρ are both fit to participant data and used to implicitly determine a number of other model parameters by imposing an equilibrium condition. This model parameterization approach leads to difficulties when implementing the profile likelihood analysis. For example, if ρ changes, then $\beta_s, \beta_r, V_s(0), V_s(0), I_s(0)$, and $I_r(0)$ also change. However, these parameters are not free to be refit as they must satisfy a equilibrium condition. Consequently, we do not include these parameters in our profile-likelihood analysis.

Furthermore, it is well known that the viral production rate p is unidentifiable when fitting viral dynamics

models to viral load data alone [4, 5]. This unidentifiability typically takes the form of a link between the viral production rate p and the baseline concentration of target cells, $T(0)$. In our modelling, we fixed $T(0) = 649500$ cells/mL to correspond to the mean concentration of CD4 T-cells measured in Stephenson et al. [6]. We refit our models to the viral load data using distinct baseline CD4 concentrations, $T_i(0) = 649500 \times 10^{-i}$ for $i = 1,2,3$ and found equivalent results to those shown in the main text, with a corresponding increase in the fit values of p.

Comparison of pharmacokinetic profiles

We considered the following two compartment pharmacokinetic (PK) model for the PGT121 dynamics

$$\left.\begin{aligned} \frac{\mathrm{d}}{\mathrm{dt}}A_1(t) &= Dose(t) - k_{12}A_1(t) + \frac{vol_2}{vol_1}k_{21}A_2(t) \\ \frac{\mathrm{d}}{\mathrm{dt}}A_2(t) &= \frac{vol_1}{vol_2}k_{12}A_1(t) - k_{21}A_2(t) - k_0A_2(t), \end{aligned}\right\} \tag{S7}$$

where the first compartment represents the dynamics of PGT121 in the circulation while the second compartment represents the peripheral tissues. We made the simplifying assumption of balanced transit between the two compartments by imposing

$$k_{21}vol_2 = k_{12}vol_1,$$

and fit $A_1(t)$ to the measured PGT121 concentrations. In related work, Cardozo-Ojeda and Perelson [4] showed that, by assuming neglecting the loss of any bnAb from the circulation during the infusion, it is possible to write the solution of the PK model independently of the ratio vol_1/vol_2. Following the argument of Cardozo-Ojeda and Perelson [4], let A_1^* and A_2^* represent the concentration of PGT121 in the circulation and peripheral tissues under the assumption that

$$\frac{\mathrm{d}}{\mathrm{dt}}A_1^*(t) = \frac{A_{max}}{T_{inf}}, \quad \text{for} \quad t \in (0, T_{inf}).$$

Using this explicit expression for A_1^* in the differential equation for A_2^*, we obtain, for $t \in (0, T_{inf})$,

$$\begin{aligned} A_1^*(t) &= \frac{A_{max_i}(t)}{T_{inf}} \\ A_2^*(t) &= \left(\frac{k_{12}A_{max_i}Vol_{1_i}}{Vol_{2_i}(k_{21}+k_0)T_{inf}}\right)\left[t - \frac{1-e^{-(k_{21}+k_0)t}}{(k_{21}+k_0)}\right]. \end{aligned}$$

Then, for t outside the dosing interval, $t \notin (0, T_{inf})$, the two-compartment PK model (S7) is a linear system of ODEs with initial conditions $A_1^*(T_{inf})$ and $A_2^*(T_{inf})$. The linear system admits the solution

$$\left.\begin{aligned} A_1^*(t) &= A_1^*(T_{inf})\left[k_i \exp\left(-\lambda_1(t-T_{inf}) + (1-k_i)\exp\left(-\lambda_2(t-T_{inf})\right]\right.\right., \\ A_2^*(t) &= A_2^*(T_{inf})e^{\left[-(k_0+k_{21})(t-T_{inf}\right]} + k_{12}\frac{vol_1}{vol_2}A_1^*(T_{inf}e^{\left[-(k_0+k_{21})(t-(T_{inf})\right]} \\ &\times\left[k_i\frac{\left(e^{\left[(k_{21}+k_0-\lambda_1)t-(T_{inf}\right]}-1\right)}{k_{21}+k_0-\lambda_1} + (1-k_i)\frac{\left(e^{\left[(k_{21}+k_0-\lambda_2)(t-T_{inf})\right]}-1\right)}{k_{21}+k_0-\lambda_2}\right] \end{aligned}\right\} \quad \text{(S8)}$$

where

$$\lambda_1 = \frac{1}{2}\left(k_{21}+k_0+k_{12}-\sqrt{(k_{21}+k_0-k_{12})^2-4k_{21}k_{12}}\right),$$
$$\lambda_2 = \frac{1}{2}\left(k_{21}+k_0+k_{12}+\sqrt{(k_{21}+k_0-k_{12})^2-4k_{21}k_{12}}\right)$$

are eigenvalues corresponding to the linear system of ODEs. The parameter k is obtained from ensuring that $A_1(t)$ is continuously differentiable at $t = T_{inf}$ and is given by

$$k = \left(\frac{k_{21}k_{12}}{\lambda_2-\lambda_1}\right)\left[\frac{e^{-(k_{21}+k_0)T_{inf}}-1}{T_{inf}(k_{21}+k_0)}+1\right]+\frac{\lambda_2-k_{12}}{\lambda_2-\lambda_1}.$$

We emphasize that the dynamics of $A_1^*(t)$ are independent of the ratio vol_1/vol_2. In Fig. F, we show a comparison of the simulated circulating PGT121 concentrations obtained by fitting the full PK model (S7) to the circulating PGT121 concentrations and simulated circulating PGT121 concentrations obtained by fitting the explicit expression $A_1^*(t)$ in (S8) to the same data. We note excellent agreement between the full model fits and the approximation $A_1^*(t)$ for all participants.

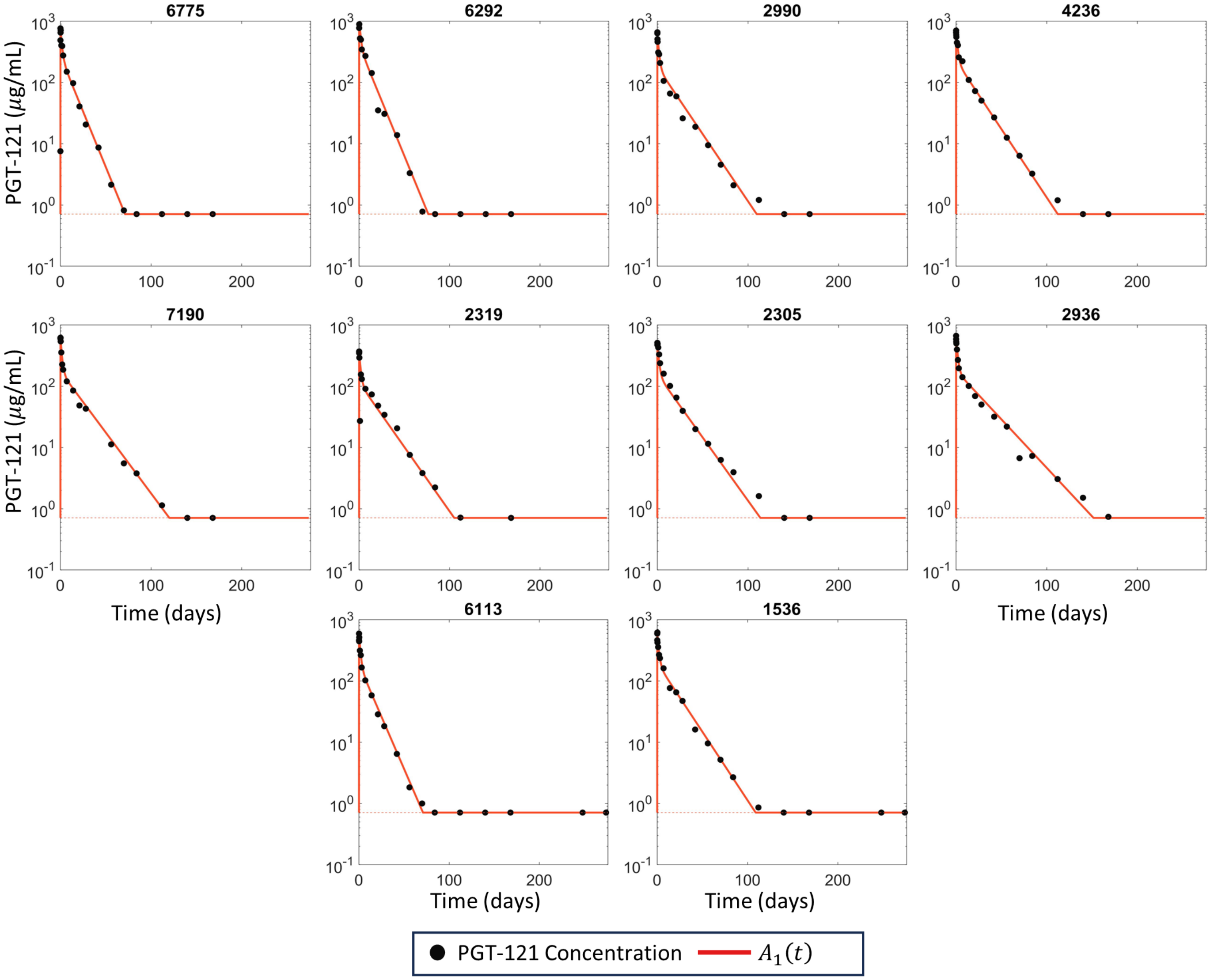

Figure A: **Fitting the two compartment PK model to participant data.** The simulated PGT121 concentration obtained using the two-compartment PK Model (S7) is shown in solid red while the PGT121 concentration is shown in black circles. The LLoQ is shown in dotted red. The best-fit parameters for each participants are given in Table B.

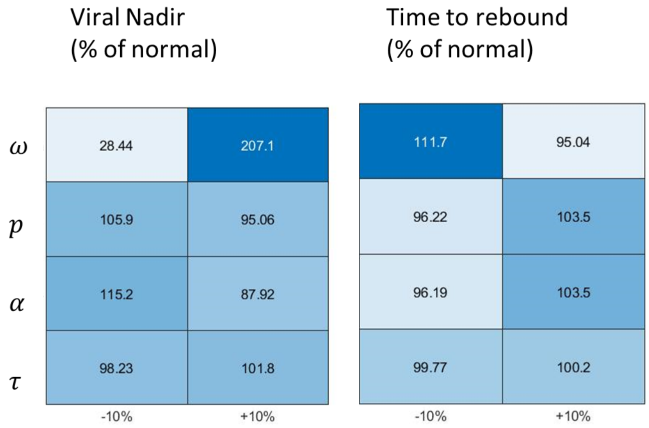

Figure B: **Local sensitivity analysis for the single population model** The left column shows the percentage change viral nadir during therapy while the right column shows the percentage change in time to viral rebound for the single population model in equations (S1) and (S4).

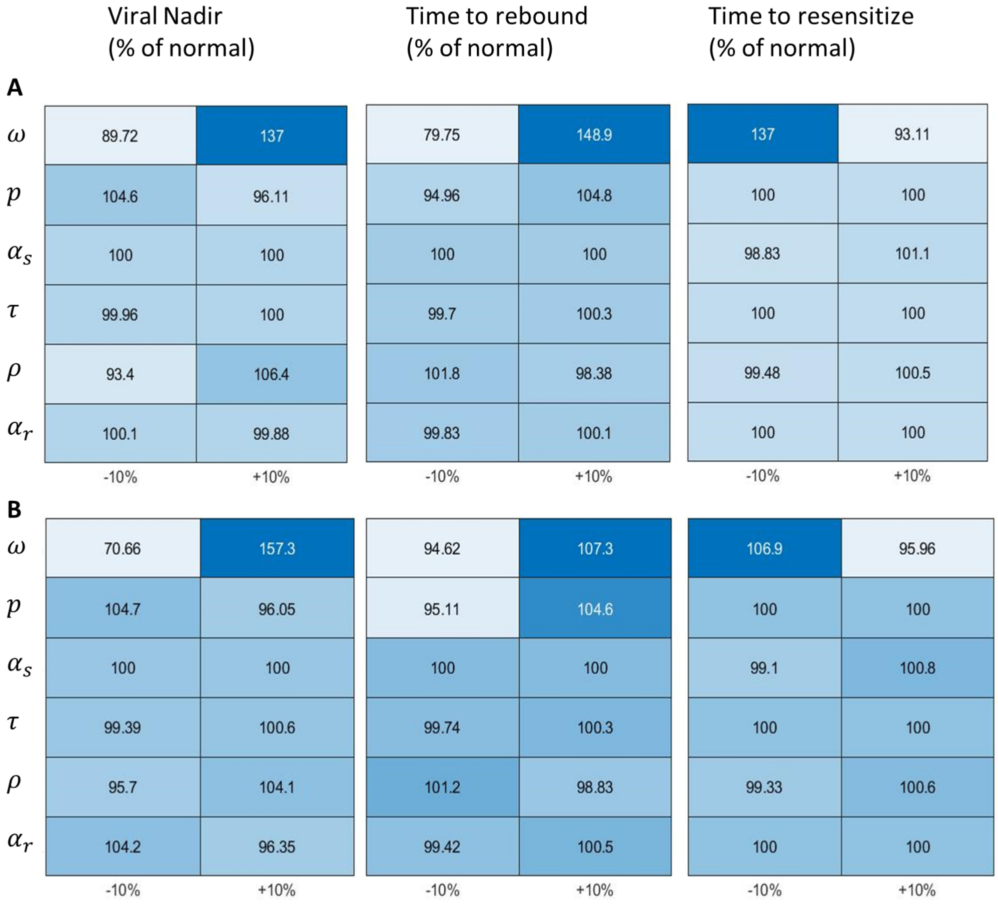

Figure C: **Local sensitivity analysis for the two population model without and with mutation** The percent changes in the viral nadir, time to rebound, and time to re-sensitization following 10% changes in each parameter are shown for the two population model without mutation in equation (S2) and (S5) in row **A**. The corresponding sensitivity analysis for the two population model with mutation in equation (S3) and (S6) are shown in row **B**.

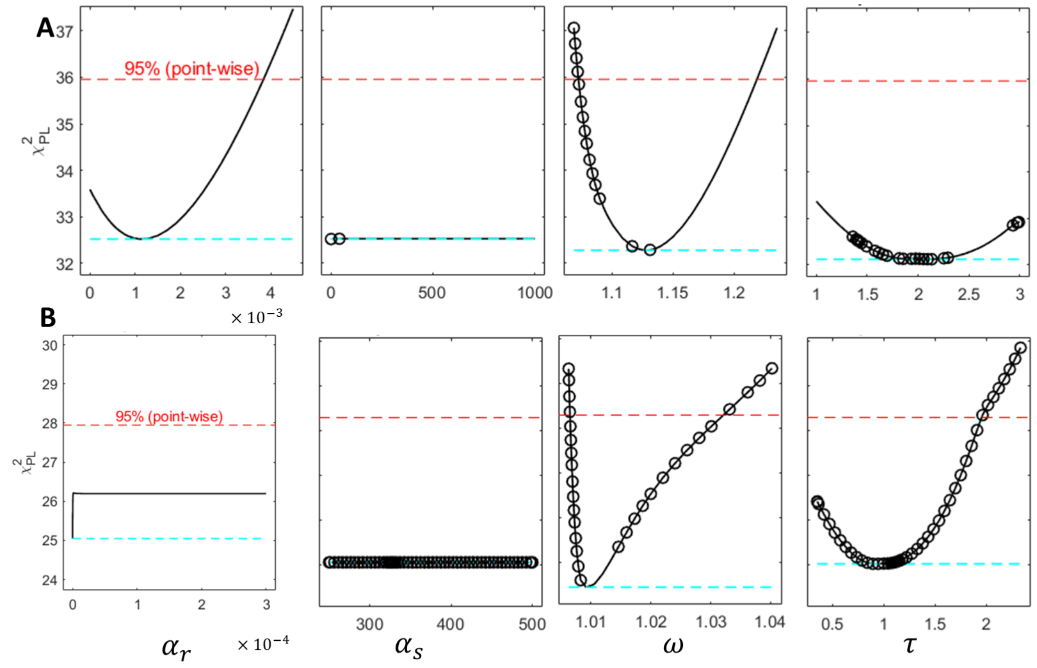

Figure D: **Profile likelihoods for participants 7190 and 2936** The profile likelihoods for the fit parameters $\alpha_r, \alpha_s, \omega$, and τ for the two strain mathematical model (Eq (S2)). The red dashed line denotes the 95% confidence interval while the black lines show the profile likelihood for each parameter. Panel A shows the profile likelihoods for participant 2936 and panel B shows the profile likelihood for participant 7190.

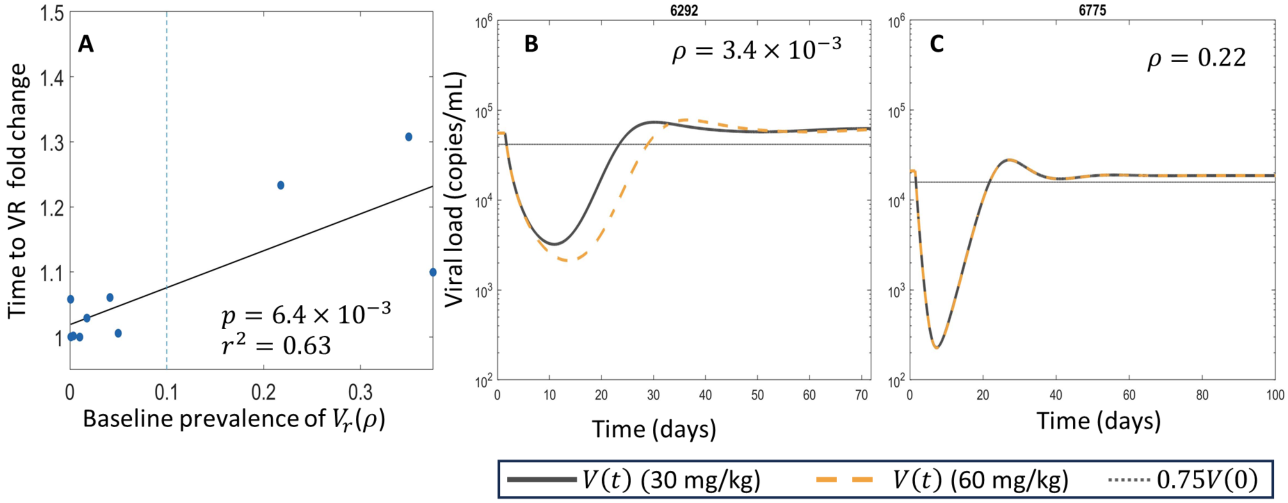

Figure E: **Baseline resistance determines sensitivity to an increased dose of PGT121.** Figure A shows the scatter plot of the fold increase in the time to viral rebound (VR) for participants in the simulated trial of 60 mg/kg PGT121 compared to 30 mg/kg PGT121 plotted against the baseline proportion of resistant virus, ρ for the two population models without mutation (Eq (S2)). In panel A, the vertical dotted line at $\rho = 0.1$ distinguishes between the groups of participants with relatively rare ($\rho < 0.1$) and common resistant virus. Figures B and C show the predicted viral load dynamics for participants 6775 and 6292 following administrations of both 30 mg/kg and 60 mg/kg of PGT121 using Eq (S2). The fit values of ρ are shown for each participant and the corresponding result for the two-population model with mutation, Eq (S3), is shown in Fig 6 of the Main Text.

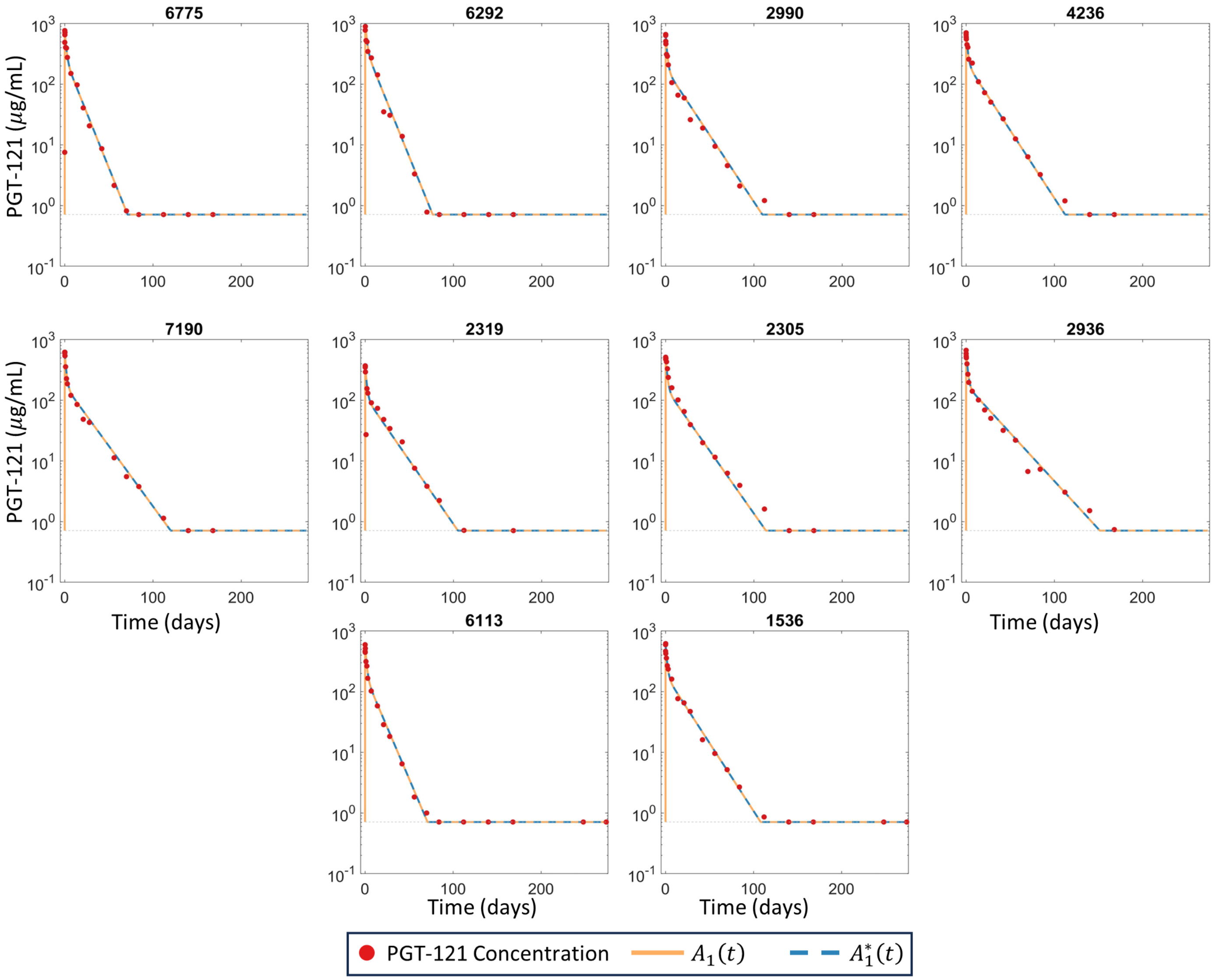

Figure F: **Comparison of pharmacokinetic models** A comparison between the best fit solution of the PK model, $A_1(t)$, and the PK approximation given by $A_1^*(t)$ in Eq (S8). The solution of the PK model, $A_1(t)$, is shown in solid orange, the approximation $A_1^*(t)$ is shown in dashed blue, and the PGT121 data is shown in solid red circles.

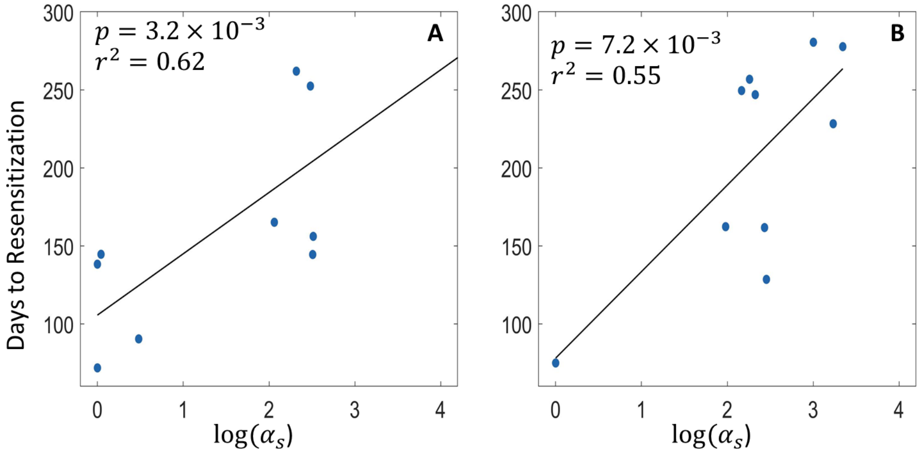

Figure G: **Time to resensitization against neutralization sensitivity** The scatter plot of the time to resensitization for participants plotted against $\log(\alpha_s)$ with the correlation coefficient and p-value. Panel A shows the results for the two population model without mutation (Eq (S2)). Panel B shows the corresponding results for the two population model with mutation, Eq (S3).

Participant	ω	p	α	τ	β	$\log_{10}(V(0))$	$\log_{10}(I(0))$
6292	1.70	8.00	2.56	1.44	1.33×10^{-4}	4.75	5.20
6775	1.21	153.70	0.03	1.27	6.91×10^{-6}	4.32	3.50
2990	1.28	8.00	0.30	1.38	1.33×10^{-4}	4.38	4.84
2305	1.06	305.40	0.01	1.48	3.48×10^{-6}	3.98	2.86
7190	1.24	85.09	0.02	1.42	1.25×10^{-5}	3.43	2.86
2319	1.02	18.91	0.08	1.17	5.62×10^{-5}	4.65	4.74
2936	1.23	13.53	0.02	1.24	7.85×10^{-5}	2.88	3.10
4236	1.46	215600.00	0.02	1.95	4.93×10^{-9}	2.54	-1.43

Table A: **Table of best fit parameters for individual participants using the single population mathematical model.** Parameter fitting results for the single population viral dynamic model equation 4 to the non-controller participants. The parameters have the following units: p (virus/cell/day); α (mL/μg); τ (days); β (mL$\times$ virus^{-1}/day); $\log_{10}(V(0))$ (log copies/mL); $\log_{10}(I(0))$ (log cells/mL).

Participant	k_{12}	k_{21}	k_0	$\log_{10}(A_{max})$
6292	0.42	0.16	0.12	4.41
6775	0.43	0.15	0.12	4.27
2990	0.47	0.14	0.07	4.20
2305	0.43	0.15	0.07	4.09
7190	0.47	0.15	0.06	4.17
2319	0.45	0.16	0.07	3.95
2936	0.47	0.14	0.05	4.21
4236	0.42	0.15	0.07	4.23
6113	0.47	0.15	0.11	4.16
1536	0.44	0.15	0.07	4.16

Table B: **Table of best fit pharmacokinetic parameters for individual participants.** Parameter fitting results for the two compartment pharmacokinetic model to PGT121 concentration data. k_{12} (1/day); k_{21} (1/day); k_0 (1/day); A_{max} (μg/mL).

Participant	ω	p	α_s	τ	ρ	α_r	β_s	β_r	$\log_{10}(V_s(0)+V_r(0))$	$\log_{10}(I_s(0)+I_r(0))$
6292	1.54	59.92	1.00	1.43	0.22	0.09	1.55×10^{-5}	7.79×10^{-6}	4.75	4.33
6775	1.13	255.90	322.20	1.42	3.49×10^{-3}	1.91×10^{-5}	4.15×10^{-6}	1.96×10^{-6}	4.32	3.28
2990	1.25	111.30	21720.00	1.79	0.01	1.33×10^{-7}	9.52×10^{-6}	3.07×10^{-6}	4.38	3.69
2305	1.33	32.10	1.00	1.41	0.35	0.07	2.86×10^{-5}	2.33×10^{-5}	3.98	3.84
7190	1.13	96.52	303.10	1.41	0.02	6.82×10^{-4}	1.10×10^{-5}	6.42×10^{-6}	3.43	2.80
2319	1.26	31.45	3.03	1.16	1.04×10^{-3}	8.61×10^{-6}	3.38×10^{-5}	5.50×10^{-6}	4.65	4.51
2936	1.01	125.30	326.90	1.11	0.05	8.19×10^{-6}	8.48×10^{-6}	8.27×10^{-6}	2.88	2.14
4236	1.31	41.97	1.10	1.79	0.38	4.97×10^{-3}	2.19×10^{-5}	1.87×10^{-5}	2.54	2.28

Table C: **Table of best fit parameters for individual participants using the two population mathematical model without mutation.** Parameter fitting results for the two population viral dynamic model in equation 5 to the non-controller participants. The parameters have the following units: p (virus/cell/day); α_s (mL/μg); τ (days); α_r (mL/μg); β_s (mL$\times$ virus^{-1}/day); β_r (mL$\times$ virus^{-1}/day); $\log_{10}(V_s(0)+V_r(0))$ (log copies/mL); $\log_{10}(I_s(0)+I_r(0))$ (log cells/mL).

Participant	ω	p	α_s	τ	ρ	α_r	β_s	β_r	$\log_{10}(V_s(0)+V_r(0))$	$\log_{10}(I_s(0)+I_r(0))$
6292	1.55	45.48	1.00	1.28	0.27	0.14	1.97×10^{-5}	1.14×10^{-5}	4.75	4.45
6775	1.21	125.20	285.20	0.94	2.13×10^{-3}	2.31×10^{-4}	8.48×10^{-6}	2.32×10^{-6}	4.32	3.59
2990	1.34	18.46	2208.00	0.62	0.28	0.08	5.14×10^{-5}	3.76×10^{-5}	4.38	4.47
2305	1.38	3.24	146.40	1.29	0.39	0.63	2.71×10^{-4}	2.29×10^{-4}	3.98	4.84
7190	1.13	100.90	211.30	1.46	0.02	1.19×10^{-3}	1.05×10^{-5}	6.43×10^{-6}	3.43	2.78
2319	1.29	8.20	1005.00	0.77	0.37	0.21	1.13×10^{-4}	9.68×10^{-5}	4.65	5.10
2936	1.01	133.30	271.20	0.85	0.05	1.96×10^{-6}	7.97×10^{-6}	7.83×10^{-6}	2.88	2.11
4236	1.49	4.73	1708.00	1.96	0.05	1.69×10^{-3}	2.19×10^{-4}	4.99×10^{-5}	2.54	3.23

Table D: **Table of best fit parameters for individual participants and the two population mathematical model with mutation.** Parameter fitting results for the two population viral dynamic model with mutation equation 5 to the non-controller participants. The parameters have the following units: p (virus/cell/day); α_s (mL/μg); τ (days); α_r (mL/μg); β_s (mL$\times$ virus^{-1}/day); β_r (mL$\times$ virus^{-1}/day); $\log_{10}(V_s(0)+V_r(0))$ (log copies/mL); $\log_{10}(I_s(0)+I_r(0))$ (log cells/mL).

Equation	ω	p	α_s	τ	ρ	α_r	aL_0	σ	β_s	β_r	$\log_{10}(V_s(0)+V_r(0))$	$\log_{10}(I_s(0)+I_r(0))$
7	1.05	16.04	511.60	1.46	-	-	0.80	0.42	6.62×10^{-5}	-	2.28	2.44
8	1.04	52.06	115.20	1.39	0.04	2.77	0.80	0.86	2.04×10^{-5}	1.82×10^{-5}	2.28	1.92
9	1.03	51.82	95.53	1.43	0.04	8.19	0.40	0.63	2.05×10^{-5}	1.84×10^{-5}	2.28	1.93

Table E: **Table of best fit parameters for participant 6113.** The parameter fitting results for each model for the long-term controller participant 6113. The N/As denote parameters that were not fit for the corresponding model. The parameters have the following units: p (virus/cell/day); α_s (mL/μg); τ (days); α_r (mL/μg); aL_0 (cells/mL/day); β_s (mL$\times$ virus^{-1}/day); β_r (mL$\times$ virus^{-1}/day); $\log_{10}(V_s(0)+V_r(0))$ (log copies/mL); $\log_{10}(I_s(0)+I_r(0))$ (log cells/mL)

Equation	ω	p	α_s	τ	ρ	α_r	aL_0	σ	β_s	β_r	$\log_{10}(V_s(0)+V_r(0))$	$\log_{10}(I_s(0)+I_r(0))$
7	1.00	21.72	288.20	1.22	-	-	0.50	0.27	4.89×10^{5}	-	2.25	2.28
8	1.01	29.10	207.80	1.17	7.18×10^{-4}	2.79	0.65	0.61	3.65×10^{-5}	3.44×10^{-5}	2.25	2.15
9	1.01	30.55	181.40	1.18	9.38×10^{-4}	59.20	0.70	0.61	3.48×10^{-5}	3.28×10^{-5}	2.25	2.13

Table F: **Table of best fit parameters for participant 1536.** The parameter fitting results for each model for the long-term controller participant 1536. The N/As denote parameters that were not fit for the corresponding model. The parameters have the following units: p (virus/cell/day); α_s (mL/μg); τ (days); α_r (mL/μg); aL_0 (cells/mL/day); β_s (mL$\times$ virus^{-1}/day); β_r (mL$\times$ virus^{-1}/day); $\log_{10}(V_s(0)+V_r(0))$ (log copies/mL); $\log_{10}(I_s(0)+I_r(0))$ (log cells/mL)

PTID	Day 0	1	2	3	7	10	14	21	28	42	56
6292[a]	4.75	4.60	4.41	4.35	3.64	3.52	3.75	4.60	4.84	4.74	4.82
6775	4.32	4.33	3.98	3.45	2.32	2.61	3.13	4.14	4.44	4.16	3.90
2990	4.38	3.99	3.71	3.54	2.93	3.54	4.01	4.19	3.90	4.19	3.83
2305	3.98	3.90	3.97	3.57	2.69	2.49	2.54	3.24	4.38	3.98	3.99
7190[b]	3.43	3.18	3.24	2.90	1.60	1.60	2.30	2.30	2.73	3.50	3.28
2319[c]	4.65	4.36	4.10	3.87	4.28	-	4.46	4.56	4.49	4.48	4.51
2936	2.88	2.77	2.28	1.90	1.60	1.60	1.90	2.15	2.74	3.24	3.12
4236	2.54	2.75	2.85	2.11	1.60	1.60	1.60	1.60	2.04	2.04	2.34

Table G: **Viral load data for individual participants.** The $\log_{10}$ viral load concentration ($\log_{10}$ copies/mL) for participants who did not exhibit long-term viral control. The top row gives the time-post-PGT121 administration, in days, that the samples were taken. The lower limit of quantification for viral load is 1.6 $\log_{10}$ copies/mL

PTID	Day 0	1	2	3	7	10	14	21	28	42	56	70	84	112	140	168	190	249	275
6113	2.28	2.32	1.95	1.70	1.60	1.60	1.60	1.60	1.60	1.60	1.60	1.60	1.60	1.60	1.60	2.79	2.20	2.20	2.70

Table H: **Viral load data for participant 6113.** The log10 viral load concentration ($\log_{10}$ copies/mL) for participant 6113. The top row gives the time-post-PGT121 administration, in days, that the samples were taken. The lower limit of quantification for viral load is 1.6$\log_{10}$ copies/mL

PTID	Day 0	1	2	3	7	10	14	21	28	43	57	70	84	113	141	190	252	283
1536	2.25	1.90	1.78	2.08	1.60	1.60	1.60	1.60	1.60	1.60	1.60	1.60	1.60	1.60	1.70	1.60	1.84	1.60

Table I: **Viral load data for participant 1536.** The log10 viral load concentration ($\log_{10}$ copies/mL) for participant 1536. The top row gives the time-post-PGT121 administration, in days, that the samples were taken. The lower limit of quantification for viral load is 1.6 $\log_{10}$ copies/mL.

[a] Sample days: 0, 1, 2, 3, 7, 10, 14, 23, 29, 45, 59
[b] Sample days: 0, 1, 2, 3, 7, 10, 14, 21, 28, 42, 57
[c] Sample days: 0, 1, 2, 3, 7, 15, 23, 30, 44, 57

PTID	Hour 0	0.5	3	6	Day 1	2	3	7	14	21	28	42	56	70	84	112	140	168	248	274
6292	3.00	2.89	2.95	-	2.72	2.70	2.54	2.43	2.16	1.55	1.49	1.14	0.52	-0.15	-0.15	-0.15	-0.15	-0.15	-	-
6775	2.69	2.89	2.82	2.85	2.61	2.60	2.44	2.18	1.99	1.61	1.31	0.94	0.33	-0.09	-0.15	-0.15	-0.15	-0.15	-	-
2990	2.80	2.82	2.71	2.66	2.49	2.46	2.32	2.03	1.82	1.77	1.42	1.28	0.98	0.66	0.32	0.08	-0.15	-0.15	-	-
2305	2.71	2.68	-	2.69	2.63	2.52	2.38	2.21	2.01	1.82	1.60	1.30	1.06	0.80	0.60	0.21	-0.15	-0.15	-	-
7190	2.78	2.79	2.79	2.73	2.55	2.36	2.27	2.08	1.93	1.69	1.63	-	1.05	0.74	0.58	0.06	-0.15	-0.15	-	-
2319	2.54	2.57	2.56	2.47	1.43	2.19	2.12	1.96	1.87	1.68	1.53	1.31	0.88	0.58	0.35	-0.14	-	-0.15	-	-
6113	2.66	2.77	2.65	2.71	2.50	2.42	2.22	2.01	1.77	1.46	1.26	0.81	0.26	0.00	-0.15	-0.15	-0.15	-0.15	-0.15	-0.15
1536	2.67	2.78	2.79	2.63	2.55	2.43	2.37	2.21	1.88	1.82	1.67	1.21	0.98	0.71	0.43	-0.07	-0.15	-0.15	-0.15	-0.15
2936	2.82	2.77	2.73	2.70	2.60	2.43	2.29	2.15	2.01	1.84	1.70	1.50	1.34	0.83	0.86	0.48	0.18	-0.15	-	-
4236	2.82	2.85	2.78	2.74	2.65	2.61	2.41	2.35	2.04	1.86	1.71	1.43	1.10	0.80	0.51	0.08	-0.15	-0.15	-	-

Table J: **PGT121 PK data for individual participants.** The $\log_{10}$ concentration of circulating PGT121 ($\log_{10}$ μg/mL) in participants. The top row gives the time-post-PGT121 administration that the samples were taken. The first 4 samples were taken within 6 hours of PGT121 administration while the remaining sample times are given in days post administration. The lower limit of quantification for the PGT121 assay is $-0.15\log_{10}$ μg/mL.

References

[1] Conway JM, Perelson AS. Residual viremia in treated HIV+ individuals. PLOS Comput Biol. 2016;12:e1004677. doi:10.1371/journal.pcbi.1004677.

[2] Borducchi EN, Cabral C, Stephenson KE, Liu J, Abbink P, Ng'ang'a D, et al. Ad26/MVA therapeutic vaccination with TLR7 stimulation in SIV-infected rhesus monkeys. Nature. 2016;540:284–287. doi:10.1038/nature20583.

[3] Kass RE, Raftery AE. Bayes factors. J Am Stat Assoc. 1995;90:773–795. doi:10.1080/01621459.1995.10476572.

[4] Cardozo-Ojeda EF, Perelson AS. Modeling HIV-1 within-host dynamics after passive infusion of the broadly neutralizing antibody VRC01. Front Immunol. 2021;12:1–20. doi:10.3389/fimmu.2021.710012.

[5] Miao H, Xia X, Perelson AS, Wu H. On identifiability of nonlinear ODE models and applications in viral dynamics. SIAM Rev. 2011;53:3–39. doi:10.1137/090757009.

[6] Stephenson KE, Julg B, Tan CS, Zash R, Walsh SR, Rolle CP, et al. Safety, pharmacokinetics and antiviral activity of PGT121, a broadly neutralizing monoclonal antibody against HIV-1: a randomized, placebo-controlled, phase 1 clinical trial. Nat Med. 2021;27:1718–1724. doi:10.1038/s41591-021-01509-0.
